# Single-nucleus profiling reveals a core disease signature and cell type–specific vulnerabilities in early Rett syndrome

**DOI:** 10.1126/sciadv.aeb4265

**Published:** 2026-06-10

**Authors:** Yan Li, Ashley G. Anderson, Guantong Qi, Sih-Rong Wu, Jean-Pierre Revelli, Hu Chen, Zhandong Liu, Huda Y. Zoghbi

**Affiliations:** ^1^ Department of Molecular and Human Genetics, Baylor College of Medicine, Houston, TX, USA.; ^2^ Genetics and Genomics program, Baylor College of Medicine, Houston, TX, USA.; ^3^ Jan and Dan Duncan Neurological Research Institute at Texas Children’s Hospital, Houston, TX, USA.; ^4^ Department of Neuroscience, Baylor College of Medicine, Houston, TX, USA.; ^5^ Department of Pediatrics, Baylor College of Medicine, Houston, TX, USA.; ^6^ Howard Hughes Medical Institute, Baylor College of Medicine, Houston, TX, USA.

## Abstract

Rett syndrome (RTT) is an X-linked neurological disorder caused by *MECP2* mutations, creating distinct cellular environments in females (mosaic) versus males (nonmosaic). Despite female patients representing most cases, how mosaicism contributes molecularly to RTT pathogenesis, particularly in presymptomatic stages, remains poorly understood. To address this question, we profiled hippocampal transcriptomes of young female and male RTT mice using bulk and single-nucleus RNA sequencing. We identified a core disease signature of consistently dysregulated genes only in MeCP2^−^ cells across RTT models. Moreover, we uncovered non–cell autonomous effects exclusively in female MeCP2^+^ excitatory neurons, suggesting that these circuits are more vulnerable early in the mosaic RTT environment. The single-nuclei data also revealed an underappreciated MeCP2^−^ interneuron subtype that had the most transcriptional dysregulation in both male and female RTT hippocampi. Together, these data highlight the different effects of MeCP2 loss on excitatory and inhibitory circuits between the mosaic and nonmosaic environments in early RTT pathogenesis.

## Teaser

Single-cell profiling of mosaic Rett hippocampus uncovers how cellular mosaicism drives vulnerabilities before symptoms emerge.

## INTRODUCTION

Rett syndrome (RTT, OMIM:312750) is a severe neurological disorder caused by loss-of-function mutations in the X-linked gene methyl-CpG binding protein 2 (*MECP2*) (*1*). Classic RTT predominantly affects females and is characterized by a period of normal development until ~6 to 18 months, followed by developmental regression. This regression is marked by loss of acquired motor, language, and social skills, development of stereotyped hand movements, irregular breathing, and intellectual disability (*2*). A key feature of RTT is the unique mosaic cellular environment in females, where random X-chromosome inactivation (XCI) results in approximately half of cells expressing either a mutant or wild-type (WT) copy of *MECP2* (*3*). This mosaicism fundamentally shapes disease presentation, as shown by the correlation between XCI skewing patterns and phenotypic severity (*4*, *5*). In contrast, males with germline *MECP2* mutations, where all cells express mutant *MECP2*, typically present with neonatal encephalopathy unless the mutation is relatively mild (*6*–*9*). The difference between female (mosaic) and male (nonmosaic) RTT raises important questions about how cellular mosaicism influences neuronal dysfunction in RTT pathogenesis and whether distinct molecular pathways emerge in these different cellular contexts.

*MECP2* encodes a methyl-cytosine binding protein (MeCP2) that is highly abundant in postnatal neurons and functions as a transcriptional regulator, whose dysfunction causes thousands of genes to be up- or down-regulated (*10*–*15*). While animal models of *Mecp2* loss-of-function mutations effectively recapitulate the human disorder (*16*–*18*), the mosaic and nonmosaic conditions present fundamentally different contexts. Female *Mecp2^+/−^* mice show a progressive disease course that parallels classic RTT, while male *Mecp2^−/y^* mice develop severe early phenotypes and die at a young age (8 to 12 weeks) (*19*, *20*). Despite these differences in disease trajectory, male mouse models have been the predominant model for most molecular studies in the field, providing a valuable system for studying the direct molecular consequences of complete MeCP2 loss. In contrast, far fewer studies have examined the molecular changes in female RTT models (*21*–*24*), leaving a gap in our understanding of how RTT disease progression unfolds in the mosaic cellular environment. To understand whether an MeCP2 null cell behaves similarly or differently in a homogeneous versus heterogeneous context requires an experimental design that compares null cells in males and females at the same chronological age, not at equivalent disease stages. While male *Mecp2^−/y^* mice show earlier symptom onset than female *Mecp2^+/−^* mice, this difference itself reflects the biological consequences of mosaicism. Comparing age-matched animals isolates the specific effects of the mosaic cellular environment on disease progression, whereas stage matching would obscure these mechanisms by selecting time points where secondary pathological processes have already diverged between the sexes.

While most molecular studies are performed at symptomatic stages of the disease, recent studies from our laboratory have highlighted the importance of studying presymptomatic time points in RTT (*25*, *26*). First, training of female RTT mice in the presymptomatic but not in the symptomatic phase can substantially delay symptom onset, suggesting a critical window for such an intervention to overcome MeCP2 loss (*26*). Second, transcriptional dysregulation precedes functional changes by several weeks in the hippocampus of adult male mice after acute deletion of *Mecp2*, showing that early transcriptional dynamics drive disease pathogenesis (*25*). These observations led us to focus on presymptomatic or early symptomatic time points at the same chronological age in both male and female mouse models to ask a series of questions as follows: Do RTT females show presymptomatic transcriptional changes? If so, are these changes shared between male and female RTT models when examined at the same age? How does the mosaic cellular environment modify the trajectory of molecular dysregulation? Last, are these transcriptomic changes specific to distinct cell types, including MeCP2^+^ and MeCP2^−^ cells in the mosaic context?

To answer these questions, we designed experiments using both bulk RNA sequencing (RNA-seq) and single-nucleus RNA-seq (snRNA-seq) to molecularly profile the hippocampus of male (NULL, *Mecp2^−/y^*) and female (HET, *Mecp2^+/−^*) RTT mice at the same chronological ages. We examined two early time points in disease progression [postnatal day 28 (P28) corresponding to 4 weeks and P45 corresponding to 6.5 weeks]. We deliberately chose age-matched rather than stage-matched comparisons to determine how the mosaic cellular environment itself influences molecular pathology, independent of downstream compensatory or secondary disease processes that emerge at different rates in males versus females. At 4 weeks, *Mecp2* transcript and protein levels become stable in the postnatal brain, and at 6.5 weeks, female RTT mice have no measurable behavioral deficits while male mice show early symptoms. We focused our transcriptional analysis on the hippocampus, a region where disruption of excitatory and inhibitory (E/I) balance, accompanied by learning and memory deficits, is among the earliest and most pronounced phenotypes in both male and female RTT mice (*19*, *27*–*32*).

Using bulk RNA-seq, we identified an early molecular disease signature shared across both male and female RTT mouse models, independent of time point. Using snRNA-seq, we profiled >120,000 nuclei, including sorted MeCP2^+^ and MeCP2^−^ neurons from female RTT hippocampus, and revealed that this early disease signature is specific to MeCP2^−^ neurons in the mosaic brain. Our single-nucleus analyses gave us the resolution to uncover shared and unique cell type–specific molecular changes in the mosaic and nonmosaic RTT hippocampus. Notably, we found widespread transcriptional changes in MeCP2^−^ and, unexpectedly, MeCP2^+^ female RTT hippocampal excitatory neurons, suggesting that excitatory neurons in the mosaic condition are highly sensitive to the altered circuit environment early in disease pathogenesis. We also identified an interneuron (IN) subpopulation (with high *Chrm2* expression) that showed a prominent transcriptional response to direct loss of MeCP2 in both male and female RTT. Together, this study uncovered an early molecular disease signature in both male and female RTT that is MeCP2 dependent and not secondary to broader circuit dysfunction. By comparing age-matched rather than stage-matched animals, we identified both shared and distinct transcriptional changes that emerge in mosaic versus nonmosaic contexts, and we revealed the earliest transcriptional changes in the presymptomatic RTT female mice. This study also provides insight into the distinct differences of cell-autonomous and non–cell autonomous changes in E/I neurons of RTT females. These data provide important neurobiological insights into the cellular and molecular drivers of pathogenesis in RTT, and are potentially relevant to other X-linked disorders.

## RESULTS

### Early disease signature in the RTT hippocampus

**Fig. 1.**
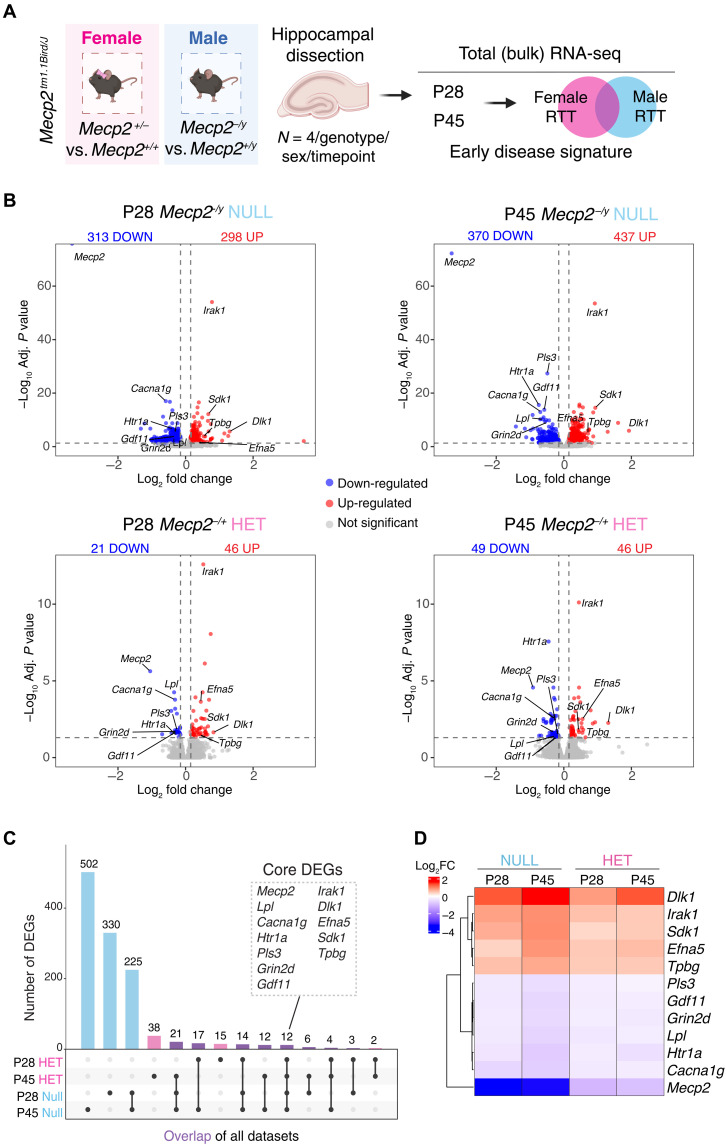
Bulk RNA-seq of hippocampal tissue in male and female RTT mice at pre- and early- symptomatic time points. (**A**) Schematic of experimental design using hippocampal tissue from male and female RTT mice along with sex-specific controls at two distinct time points (P28 and P45) to examine early transcriptional disease signatures via bulk RNA-seq. Created in BioRender. Li, Y. (2026) https://BioRender.com/d8t1ywf. (**B**) Volcano plots showing significant DEGs that are up-regulated (red) or down-regulated (blue) at both time points in male NULL (top) or female HET (bottom) compared to their sex- and age-matched control WT samples using a |log_2_FC| > 0.15 and adjusted *P* value <0.05 cutoff for all comparisons. (**C**) Upset plot showing the overlap of DEGs across time points and genotypes with 12 core DEGs highlighted. (**D**) Heatmap showing the log_2_FC of core RTT DEGs at each time point within NULL and HET samples.

Since male and female RTT mice have distinct time courses for disease progression, we examined whether there is a shared disease signature that is consistent between sexes and can be detected early in disease pathogenesis. We first compared the transcriptional changes in the female and male RTT hippocampus using bulk RNA-seq in a mouse model with a null *Mecp2* allele (*Mecp2^tm1.1Bird/J^*) (*16*), which produces no MeCP2 protein and shows hippocampal phenotypes at 6 weeks in NULL mice and at 12 weeks in HET mice (*16*, *19*). To avoid the confounds of phenotypic and secondary changes between male and female RTT mice, we used early time points in disease course (P28 and P45) and examined differentially expressed genes (DEGs) between genotypes within each sex and time point (Fig. 1A and table S1). Principal components analysis of all samples together showed that samples clustered primarily by sex (fig. S1A). Male samples showed clear separation by time point and genotype, while female samples were more interspersed (fig. S1A). We found that NULL mice had ~three times more DEGs at each time point compared to HET mice (Fig. 1B). The magnitude of expression level changes was also greater in NULL compared to HET mice at both time points (Fig. 1B). In NULL samples, the number of both up- and down-regulated DEGs increased progressively from P28 (313 down, 298 up; total 611 DEGs) to P45 (370 down, 437 up; total 807 DEGs). Despite showing no measurable phenotypes at these early time points, female HET mice exhibited detectable transcriptional changes at P28 (21 down, 46 up; total 67 DEGs) and P45 (49 down, 46 up; total 95 DEGs).

By overlapping the DEGs across both genotypes and time points, we found 12 DEGs (termed “core RTT DEGs”), including *Mecp2*, that were persistently dysregulated in HET and NULL samples in the same direction, indicative of an early disease molecular signature across RTT models (Fig. 1C). Five genes were consistently up-regulated (*Dlk1*, *Irak1*, *Sdk1*, *Efna5*, and *Tpbg*) and seven genes were consistently down-regulated (*Pls3*, *Gdf11*, *Grin2d*, *Lpl*, *Htr1a*, *Cacna1g*, and *Mecp2*) (Fig. 1D and fig. S1B). Seven of 12 genes (*Cacna1g*, *Htr1a*, *Efna5*, *Tpbg*, *Grin2d*, *Sdk1*, and *Pls3*) are involved in synapse-related gene ontology categories. Moreover, the magnitude of change in core RTT DEGs increased as the animals aged, particularly within NULL mice (Fig. 1D).

Our laboratory has previously shown that *Gdf11* is positively regulated by MeCP2 and is sensitive to *Mecp2* level changes (*33*), which we confirm can be detected as early as 4 weeks in both male NULL and female HET tissue. The biological significance of these core RTT DEGs is supported by their overlap with our previous study examining the molecular cascade following the acute loss of MeCP2 within the hippocampus of adult male mice using the *Mecp2^tm1.1Jae^* floxed allele [adult knockout (KO)] (*17*, *25*). We found that 11 of our core RTT DEGs (all but *Irak1*) overlapped in the same direction with DEGs from acute loss of MeCP2 between 4 and 8 weeks when MeCP2 levels were stably depleted (17.1-fold enrichment, *P* < 1.0 × 10^−10^, hypergeometric test) (fig. S1C). The unique observation that *Irak1* is only changed in the constitutive KO but not after acute *Mecp2* loss in adult mice suggests that changes in *Irak1* may be due to either a developmental effect of *Mecp2* on this gene or a local effect of the engineered *Mecp2* allele, given that *Irak1* is located only 3 kilobases distal to the replacement cassette (*16*, *34*). The magnitude of change in expression also progressively increased over time for core RTT DEGs in the adult KO datasets (fig. S1D). To further validate our core RTT signature, we compared our 12 genes with previously published hippocampal studies. Eleven of 12 genes (91.7%) were also dysregulated in the Osenberg *et al.* (*35*) hippocampal *Mecp2* knockdown study, and all 12 core genes showed identical directional changes in the study by *Petazzi et al.* (*36*) (fig. S1E). Further, all 12 core genes were independently identified as brain-specific common core changes in the Trostle *et al.* (*37*) meta-analysis across multiple RTT studies (hypergeometric test, *P* < 2.2 × 10^−16^). Beyond the core genes, we also compared our P45 bulk RNA-seq data with previously published hippocampal datasets and meta-analyses [Osenberg *et al.* (*35*), Petazzi *et al.* (*36*), Baker *et al.* (*38*), and Trostle *et al.* (*37*)] and found high concordance between our data and these independent studies (fig. S1, F to I). Together, these data identify a shared core molecular disease signature in both female and male RTT models early in the disease course before female RTT mice develop neurological symptoms (*19*), suggesting that these transcriptional changes represent an important step in disease pathogenesis rather than being secondary to the RTT phenotype. Furthermore, the data confirm that our datasets reliably detected some of the established molecular features of RTT pathology.

### Transcriptional changes in male and female RTT hippocampi are restricted to mature cell types

**Fig. 2.**
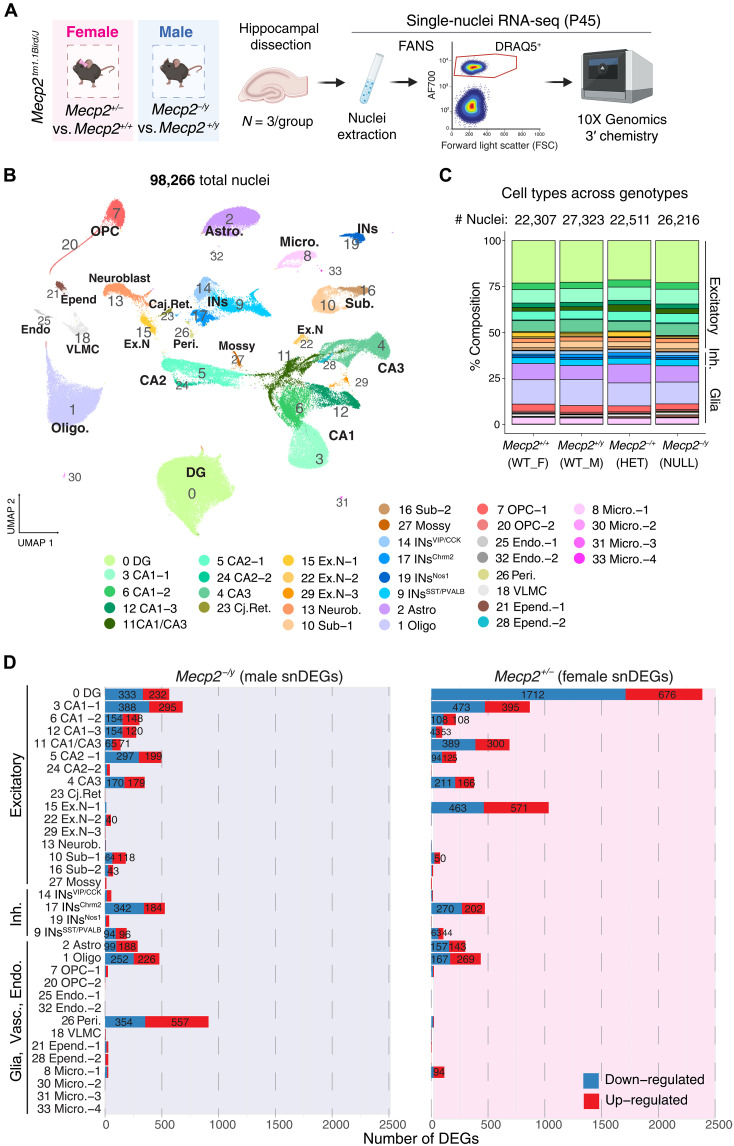
snRNA-seq of hippocampi from male and female RTT mice. (**A**) Schematic of experimental design of snRNA-seq using hippocampi from male and female RTT mice at P45 time point. Created in BioRender. Li, Y. (2026) https://BioRender.com/d8t1ywf. (**B**) Uniform Manifold Approximation and Projection (UMAP) of all hippocampal nuclei passing quality control across genotypes and annotated by cell type (DG, dentate; Ex.N, excitatory neuron; IN, interneurons; Sub, subiculum; Cj. Ret, Cajal-Retzius; Astro, astrocyte; Oligo, oligodendrocyte; OPC, oligodendrocyte precursor cell; Endo, endothelial; Peri, pericyte; VLMC, vascular and leptomeningeal cells; Epend, ependymal; micro, microglia). (**C**) Bar graph showing the percent cell type composition across each genotype and counts of total nuclei (no significant changes in cell type proportion; see Materials and Methods). (**D**) Bar graphs showing the number of DEGs (|log_2_FC| > 0.15 and adjusted *P* value <0.05 was used to filter for all cell types) either up-regulated or down-regulated within each cluster in male (left) and female (right) RTT cell types.

MeCP2 is broadly expressed in various cell types throughout the brain, but its protein levels vary among different cell populations (*39*–*41*). Furthermore, cell type–specific deletion of *Mecp2* results in distinct phenotypic patterns in mouse models (*42*–*45*). To investigate whether different cell types exhibit differential sensitivity to MeCP2 loss, we analyzed gene expression changes using snRNA-seq in the hippocampus of both male and female RTT mice. We isolated hippocampal nuclei via fluorescence-activated nuclei sorting (FANS) from *Mecp2^−/y^* (NULL) and *Mecp2^+/−^* (HET) animals at P45, matching the time point in our bulk RNA-seq dataset (Fig. 2A). We targeted 10,000 nuclei per sample (*N* = 3 per group, 12 samples total), and 98,266 nuclei passed quality control criteria (fig. S2A). We did not find differences in total RNA count across genotypes (fig. S2A). In the snRNA-seq dataset, we observed more reads mapping to the *Mecp2* transcripts in NULL and HET samples. However, we confirmed that none of the reads mapped to the deleted exons 3 and 4 region in the *Mecp2^tm1.1Bird^* allele, indicating the absence of functional *Mecp2* transcript (fig. S2C). Unsupervised clustering detected a total of 34 clusters (Fig. 2B). We annotated the clusters by comparing their top unique marker genes (table S2) to the known marker genes of hippocampal cell types from previous single-cell studies (Fig. 2B and fig. S2D) (*46*, *47*). We did not observe differences in cell type composition across genotypes (Fig. 2C, fig. S2B, and table S3), validating previous findings that loss of MeCP2 neither alters cell identity nor leads to cell death (*48*). To identify cell type–specific DEGs, we performed differential expression analysis within each cluster comparing female and male RTT models to their respective sex- and littermate-matched controls (*Mecp2^+/−^* versus *Mecp2^+/+^* females or *Mecp2^−/y^* versus *Mecp2^+/y^* males) (Fig. 2D and table S4). Both up-regulated and down-regulated single-nucleus DEGs (snDEGs) were enriched in multiple cell types in male and female RTT mice, including excitatory hippocampal neuronal clusters [dentate (DG), CA1, CA2, and CA3], distinct IN clusters (INs^CHRM2^ and INs^SST/PVALB^), and specific glial clusters (oligodendrocytes and astrocytes) (Fig. 2D). We detected sex-specific DEG signatures in two cell types: Pericytes (cluster 26) were specific to NULL samples, while excitatory neuronal subtype 1 (Ex. N-1, cluster 15) was specific to HET samples. Notably, immature cell types, such as oligodendrocyte precursor cells (OPCs) or neuroblasts, did not have many transcriptional changes, highlighting the important role of MeCP2 in mature neuronal cell types (Fig. 2D) (*17*, *25*). Together, these results demonstrate that most transcriptional changes occur in mature hippocampal cell types in both males and females.

### Comparison of cell type–specific transcriptional changes between male and female RTT models

**Fig. 3.**
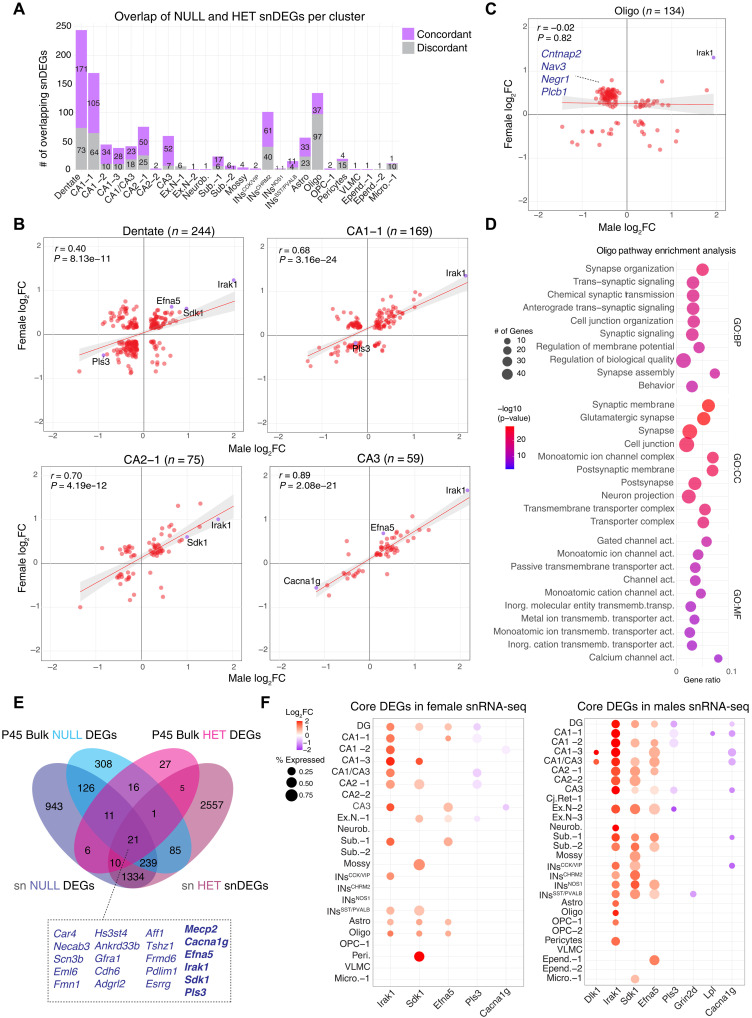
Shared and unique transcriptional signatures across cell types in hippocampi of male and female RTT mice. (**A**) The number of snDEGs overlapping between female and male RTT samples across all clusters. (**B**) Correlation of log_2_FC values in shared DEGs between male and female RTT samples in the dentate (DG), CA1-1, CA2-1, and CA3 clusters. (**C**) Correlation of log_2_FC values in shared male and female DEGs in oligodendrocytes. (**D**) GO enrichment analysis of genes that are up-regulated in female but down-regulated in male oligodendrocytes. (**E**) Venn diagram showing the overlap of bulk RNA-seq DEGs and unique snDEGs between male (blue) or female (pink) RTT samples. (**F**) Bubble plot showing core DEGs that are also snDEGs across distinct cell types in female (left plot) and male (right plot) RTT hippocampus. Only clusters with snDEGs detected are shown on the plots.

Since females with mosaic *Mecp2* loss show slower disease progression than males with complete *Mecp2* loss, previous studies comparing male and female models have usually been conducted at different ages. Here, we identify shared disease signatures between age-matched males and females at cell type resolution. We examined the shared molecular signatures of NULL and HET samples within each cell type by overlapping the snDEGs within each cluster and performing hypergeometric tests separately for up-regulated and down-regulated gene sets within each cluster (Fig. 3A and table S5). We found that most snDEGs overlapping within each cluster had concordant gene expression directionality between male and female RTT models. We also found that the magnitude of shared DEGs correlated between HET and NULL samples within nearly all clusters, particularly the excitatory neuronal clusters within the CA1, CA2, and CA3 regions (Fig. 3B). DG excitatory neurons were the least concordant excitatory neuron population (Fig. 3B). Among nonneuronal cell types, oligodendrocytes showed the largest number of shared DEGs between males and females, yet those overlapping DEGs lacked a positive correlation in direction (Fig. 3C). To examine this further, we aggregated the snRNA-seq data into pseudobulk profiles and confirmed that the majority (>50%) of the pseudobulk oligodendrocyte DEGs did not show a consistent direction of change between sexes (fig. S3A). We further performed a deconvolution analysis on our bulk RNA-seq datasets at both time points (fig. S3B) and found a positive correlation between male and female DEGs across all cell types except oligodendrocyte. At P45, oligodendrocytes do not show a significant positive correlation (*r* = 0.267, *P* = 0.301) between males and females. Among oligodendrocyte DEGs concordantly regulated, we observed a strong correlation between sex (*r* = 0.916, slope = 0.52; fig. S3C), indicating that concordant genes show consistent directionality but with reduced effect sizes in females, indicating a buffering effect in the female, mosaic environment. Comparing directional concordance rates across cell types revealed that males showed significantly lower concordance between oligodendrocyte and other cell types than females (*P* < 0.001, Wilcoxon test; fig. S3D). While some cell types in males, including CCK^+^ VIP^+^ INs, Chrm2^+^ INs showed only 50 to 60% concordance, most cell types showed more than 75% concordance (fig. S3D). Pathway enrichment analysis of these 97 oligodendrocyte DEGs showed enrichment for Gene Ontology (GO) terms such as synapse organization and synaptic signaling pathways (Fig. 3D). Together, these convergent lines of evidence demonstrate that oligodendrocytes exhibit differential transcriptional responses between sexes, suggesting sensitivity to the cellular environment, although we cannot exclude potential contributions from disease stage differences between male and female mice.

### Cross-platform validation reveals enhanced cellular resolution and preserved core disease signature across mosaic and nonmosaic tissue

We were surprised to find more snDEGs across cell types in female RTT mice compared to males, which is different from our bulk RNA-seq results where males showed more DEGs. Comparing all four datasets (bulk NULL, bulk HET, snNULL, and snHET) revealed 21 genes across all conditions (hypergeometric test, *P* = 3.73 × 10^−11^; Fig. 3E), including 6 of the 12 core RTT DEGs identified earlier, further validating these as robust disease signatures. In addition, male datasets showed substantial platform concordance with 399 overlapping genes between bulk and snRNA-seq (hypergeometric test, *P* = 5.54 × 10^−141^), while female datasets showed 37 overlapping genes (*P* = 6.42 × 10^−05^; Fig. 3E). To investigate whether these snDEGs represent true disease signatures rather than technical artifacts, we performed multiple validation analyses. First, we correlated the snDEGs of both female and male RTT animals with the bulk RNA-seq DEGs from male NULL tissue (P45 dataset) and found a significant, positive Pearson correlation between both male (*r* = 0.619, *P* = 4.402042 × 10^−136^) and female (*r* = 0.565, *P* = 2.405773 × 10^−76^) snDEGs with bulk RNA-seq DEGs (fig. S3, E and F), confirming that the snDEGs reflect genuine biological changes. Second, we aggregated all cell types into pseudobulk samples and compared them to our age-matched bulk RNA-seq data. Despite differences in platform and library chemistry, we found a high correlation *r* = 0.89 between the shared pseudobulk NULL snDEGs and bulk DEGs (slope ≈ 1; fig. S3H), demonstrating the robustness of our snRNA-seq data. Third, comparing male and female DEGs at the pseudobulk level revealed significant correlation (*r* = 0.632, *P* < 0.001; fig. S3I), indicating a shared disease signature across sexes at this resolution. We also examined the distribution of snDEGs across cell types and found that most were cell type specific, appearing in only one cluster (fig. S3G). Notably, snDEGs appearing in multiple clusters (>3) showed higher overlap with bulk RNA-seq DEGs (fig. S3G), suggesting that bulk RNA-seq predominantly captures widespread transcriptional changes while missing cell type–specific alterations. Together, these data suggest that snRNA-seq might be more sensitive in capturing early transcriptional changes particularly in female RTT tissue, as cell type–specific changes might be masked in bulk samples.

We next examined the core RTT DEGs in snRNA-seq data, where we captured transcripts of nine of them (all except for *Gdf11*, *Htr1a*, and *Tpbg*)*.* This is likely due to the generally low capture rate of snRNA-seq where ~30% of transcripts are captured in any given cell (fig. S2E) (*49*); however, we did detect six core RTT DEGs (including *Mecp2*) in our snRNA-seq data (Fig. 3, E and F). These snDEGs were differentially expressed in the same direction as in the bulk dataset. Core RTT DEGs identified from our bulk RNA-seq data did not have a strong cell type–specific expression pattern, except for *Grin2d*, an *N*-methyl d-aspartate receptor (NMDAR) subunit known to be enriched in INs (fig. S2E). Male RTT snRNA-seq samples had more core DEGs in neurons compared to females, primarily in excitatory neurons of DG, CA1, CA2, and CA3 clusters. Eight core DEGs (*Dlk1*, *Irak1*, *Sdk1*, *Efna5*, *Pls3*, *Grin2d*, *Lpl*, and *Cacna1g*) were differentially expressed in male E/I neuronal clusters compared to only five core DEGs (*Irak1*, *Sdk1*, *Efna5*, *Pls3*, and *Cacna1g*) in females. Females had more core DEGs up-regulated in glial populations (*Irak1*, *Sdk1*, and *Efna5*) compared to males (*Irak1*) (Fig. 3F). These cell type–specific changes underscore the importance of studying disease signatures using multiple transcriptomic approaches. To further investigate the effects of female mosaicism, we examined the transcriptional changes of MeCP2^+^ and MeCP2^−^ neurons separately.

### Single-nuclei analysis of MeCP2^+^ and MeCP2^−^ neurons in mosaic RTT females

**Fig. 4.**
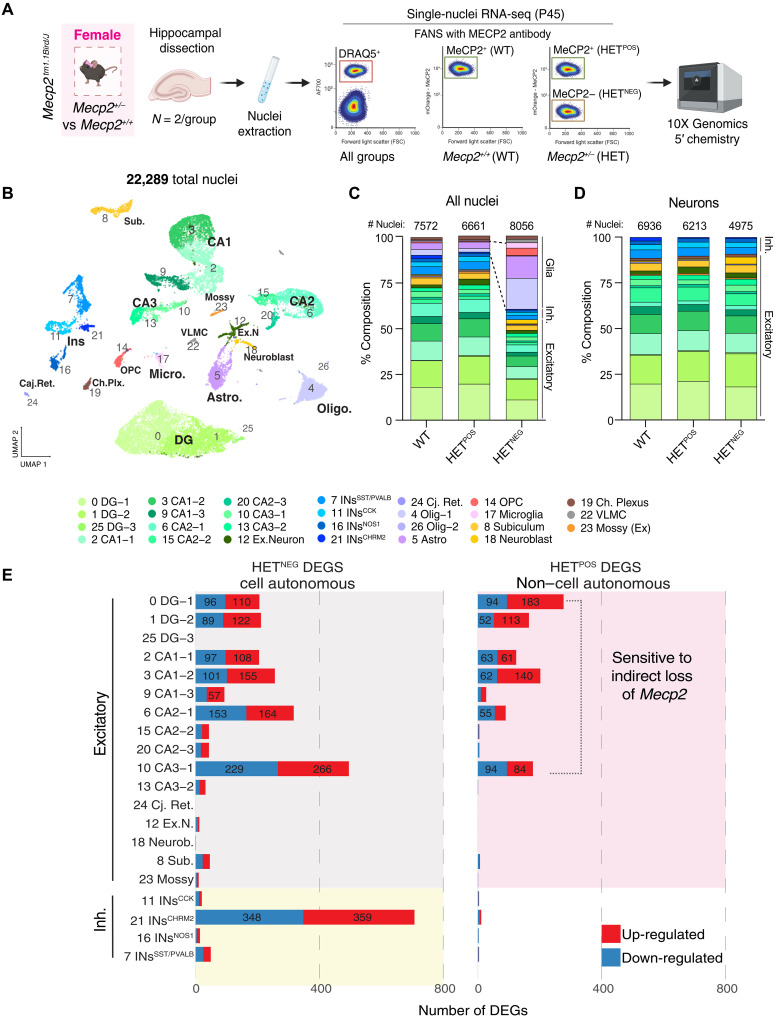
snRNA-seq of MeCP2^+^ and MeCP2^−^ cells within the mosaic hippocampus of RTT female mice. (**A**) Schematic of the experimental design using FANS to isolate nuclei from female WT and *Mecp2^+/−^* (HET) mice and then using an anti–MeCP2–phycoerythrin–conjugated antibody to capture MeCP2^+^ (WT, HET^POS^) and MeCP2^−^ (HET^NEG^ only) cells. We used the 10X 5′ chemistry kit to capture nuclei and sequence transcripts. Created in BioRender. Li, Y. (2026) https://BioRender.com/d8t1ywf. (**B**) UMAP of all female WT, HET^POS^, and HET^NEG^ nuclei that passed quality control parameters as in Fig. 2. (**C**) The percent composition within all three samples across all cell types (nonneuronal clusters 4, 14, 17, and 22 showed different proportions in different groups) and (**D**) neurons specifically. (**E**) Bar charts showing the number of DEGs (|log_2_FC| > 0.15 and adjusted *P* value <0.05) either up-regulated or down-regulated within each cluster in HET^NEG^ (left, cell autonomous changes) and HET^POS^ (right, non–cell autonomous changes) in the RTT mosaic hippocampus.

We used an antibody-based sorting strategy to identify MeCP2^+^ and MeCP2^−^ nuclei in the hippocampus of female HET and WT mice at the same time point (P45) as the previous bulk and snRNA-seq datasets (Fig. 4A and fig. S4A). Nuclei were sorted into the following three groups for downstream analyses: (i) WT (*Mecp2^+/+^*, MeCP2^+^ sorted), (ii) HET^POS^ (*Mecp2* ^+/−^, MeCP2^+^ sorted), and (iii) HET^NEG^ (*Mecp2* ^+/−^, MeCP2^−^ sorted). Using similar quality control parameters as the first dataset (fig. S4B), we analyzed 22,289 nuclei (~7 to 8 K nuclei per group) and found that unsupervised clustering separated the nuclei by cell types (Fig. 4B and fig. S4D). We observed an increase in glial cell types in the HET^NEG^ group compared to WT and HET^POS^ groups (Fig. 4C and table S3). This is due to MeCP2 being more abundant in neurons compared to glia (*40*). Glial populations predominantly fell into the negative gate for the HET^NEG^ group when using NULL cells (from male) as the control. As a result, we captured more glial populations in the HET^NEG^ group. Therefore, we subset neuronal clusters and found no neuronal cell type composition differences across groups (Fig. 4D and fig. S4C). We then focused our downstream analyses specifically on neuronal cell types.

We examined the cell type–specific transcriptional changes in HET^NEG^ and HET^POS^ groups within each cluster by comparing HET^POS^ or HET^NEG^ neurons to WT neurons (Fig. 4E and table S4). First, we found that HET^NEG^ neurons had ~three times more unique snDEGs (1787) compared to HET^POS^ snDEGs (647) (Fig. 4E). We also found that the magnitude of differential expression (average abs_Log_2_FC) was higher in HET^NEG^ DEGs compared to HET^POS^ DEGs across all clusters (fig. S4F). In HET^NEG^ neurons, we found snDEGs in both E/I hippocampal neurons, whereas HET^POS^ snDEGs were almost exclusive to excitatory neurons of the DG, CA1, CA2, and CA3 (Fig. 4E). We examined the extent to which cell-autonomous and non–cell autonomous transcriptional changes are shared across neuronal subtypes. Only 12.6% of cell autonomous DEGs were shared across three or more clusters, and 17.8% of non–cell autonomous DEGs were shared across three or more clusters (fig. S5A). This moderate level of sharing suggests that both HET^POS^ and HET^NEG^ neurons exhibit predominantly cell type–specific responses.

**Fig. 5.**
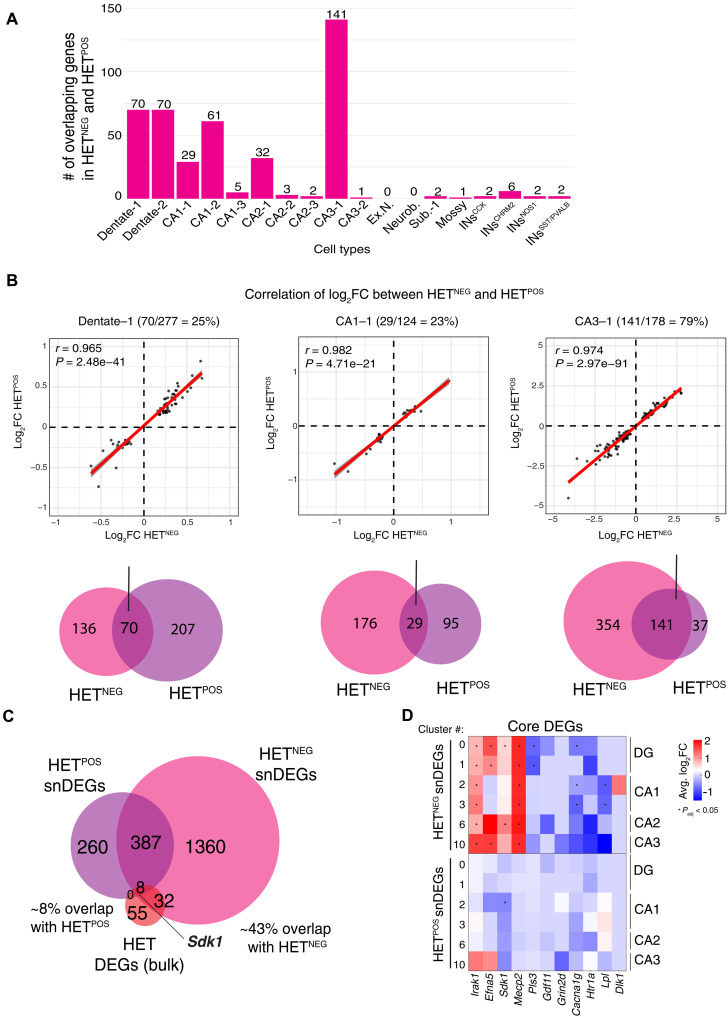
Cell type–specific transcriptional changes in the HET^POS^ and HET^NEG^ cells. (**A**) Bar plot showing the number of overlapping snDEGs between neuronal clusters of HET^POS^ and HET^NEG^ samples. (**B**) Venn diagram showing overlap between Dentate-1 (hypergeometric test, *P* = 4.97 × 10^−60^), CA1-1 (hypergeometric test, *P* = 2.14 × 10^−24^), and CA3-1 (hypergeometric test, *P* = 1.89 × 10^−164^). Correlation plots comparing the log_2_FC of snDEGs that overlapped between HET^POS^ and HET^NEG^ samples within the Dentate-1 (DG-1), CA1-1, and CA3-1 clusters. (**C**) Venn diagram showing the overlap of bulk RNA-seq DEGs from female HET samples with unique snDEGs from HET^POS^ and HET^NEG^ samples. (**D**) Heatmap of log_2_FC of core DEGs found in the HET^POS^ and HET^NEG^ excitatory clusters (* = *P*_adj_ < 0.05).

To determine whether changes in HET^POS^ neurons represent a response to the disease or a unique response potentially compensating for the disease, we compared the number of snDEGs that overlapped in excitatory clusters between HET^NEG^ and HET^POS^ neurons (Fig. 5A). Hypergeometric testing confirmed that the observed overlaps significantly exceeded expectations in all excitatory clusters (table S6). We found that ~25 to 40% of HET^POS^ snDEGs in excitatory neurons overlapped with HET^NEG^ snDEGs, and these overlapping snDEGs showed a significant strong correlation in DG, CA1, CA2, and CA3 clusters (Fig. 5B). This indicates that, at least among the shared genes, most non–cell autonomous responses are changing in the same direction. The CA3-1 cluster had the highest overlap between groups, with ~79% of HET^POS^ snDEGs overlapping and significantly correlated with HET^NEG^ snDEGs in CA3-1 (Fig. 5B, *P* = 1.89 × 10^−164^). We then examined the GO categories of the CA3-1 shared genes (fig. S5B). Up-regulated genes were enriched in pathways such as membrane potential, potassium ion transport, as well as nicotinamide adenine dinucleotide and glucocorticoid metabolic processes. Down-regulated shared genes were enriched in pathways such as synapse assembly, postsynaptic organization, and regulation of synaptic transmission (fig. S5B). CA3 neurons are unique compared to other hippocampal cell types as they form more intraregional connections within CA3 (*50*). While we observed more DEGs in CA1 and DG HET^POS^ neurons, CA3 exhibited a higher degree (CA3: 79%, DG: 25%, and CA1: 23%) of concordant changes between HET^POS^ and HET^NEG^ neurons. This pattern of shared transcriptional changes may reflect the extensive local connectivity within CA3, potentially facilitating non–cell autonomous influences from CA3 MeCP2^−^ to MeCP2^+^ neurons. Together, these data show that excitatory neurons in the female RTT model are particularly sensitive to the cellular environment created by XCI mosaicism in presymptomatic stages.

### The core molecular disease signature in female RTT is driven by cell-autonomous loss of MeCP2

To examine how the HET^POS^ and HET^NEG^ transcriptional changes in our single-nucleus data contributed to the female bulk RNA-seq disease signature, we overlapped the unique snRNA-seq DEGs from HET^NEG^ and HET^POS^ samples with our HET bulk RNA-seq DEGs (at P45 time point) (Fig. 5C). We found that the bulk DEGs overlapped with snDEGs either exclusive to HET^NEG^ (~43%) or overlapping between HET^NEG^ and HET^POS^ snDEGs (~8%). Notably, no bulk DEGs overlapped exclusively with HET^POS^ snDEGs (Fig. 5C). Compared to bulk RNA-seq, ~7 to 19 times more DEGs were captured in our single-nuclei analysis, further supporting that bulk RNA-seq of mosaic tissue masks many cell-autonomous and non–cell autonomous molecular changes. We found 11 of 12 core DEGs (all except *Tpbg*) were captured in our MeCP2-sorted snRNA-seq dataset, likely because of technical differences in 5′ versus 3′ capture of transcript (only 8 of 12 core DEGs are captured in the previous snRNA-seq dataset). Again, we found that core RTT DEGs were expressed broadly across various cell types in this dataset (fig. S4E). We next examined whether the 11 core RTT DEGs were differentially expressed in HET^NEG^ and HET^POS^ excitatory neuron clusters of the DG, CA1, CA2, and CA3 (Fig. 5D). We found that most core RTT DEGs were specific to HET^NEG^ neuronal clusters and changed in the same direction as our previous bulk and snRNA-seq datasets. *Sdk1* was significantly down-regulated in one HET^POS^ cluster of the CA1 region (CA1-1), in the opposite direction from our other datasets, suggesting that molecular compensation of a core DEG occurs in a subpopulation of excitatory neurons (Fig. 5D). Overexpression of *Sdk1*, which encodes Sidekick-1, a member of the immunoglobulin superfamily of adhesion molecules, has been associated with increased stress susceptibility in the ventral hippocampus (*51*). *Sdk1* also plays a critical role in synapse formation and neuronal connectivity by mediating specific synaptic connections through homophilic adhesion (*52*, *53*). This may explain why *Sdk1* dysregulation in HET^NEG^ neurons could disrupt proper circuit connectivity and affect HET^POS^ neurons. Together, these data show the core molecular signature is largely driven by direct loss of *Mecp2*.

### Cell autonomous gene expression changes in MeCP2^−^ trilaminar neurons suggest their vulnerability in RTT

**Fig. 6.**
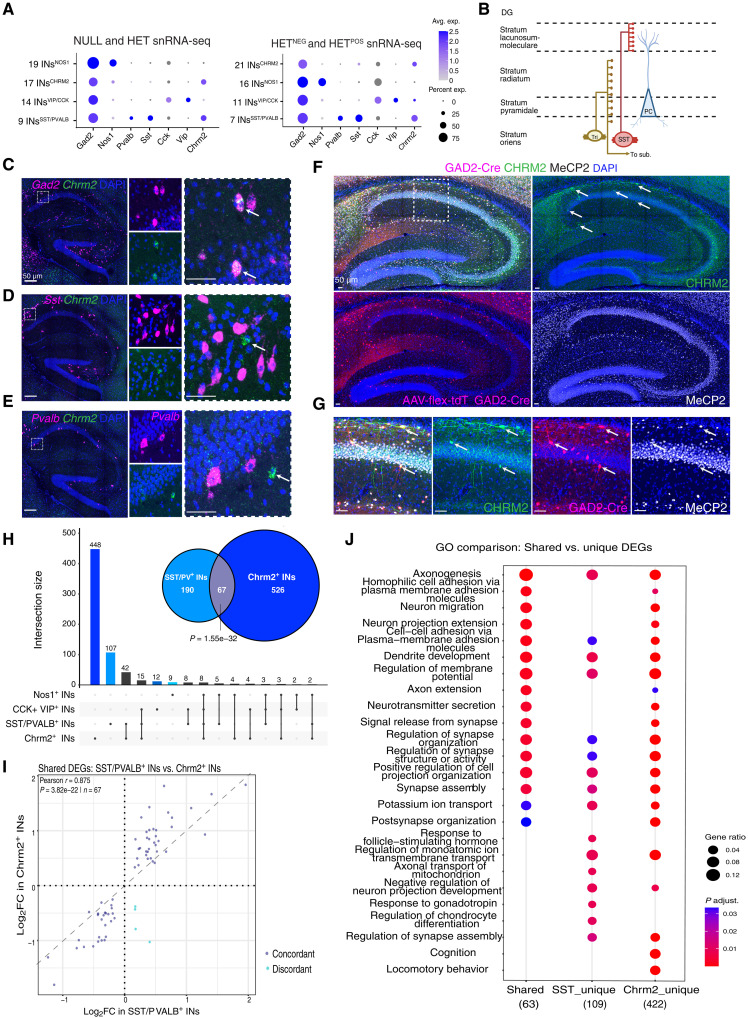
Validation of trilaminar IN molecular markers and spatial location in the hippocampus. Dot plots of top IN cluster molecular markers in (**A**) both male and female hippocampus datasets (left) and female MeCP2-sorted dataset (right). (**B**) Schematic diagram of Chrm2^+^ trilaminar INs anatomical location and morphology in the hippocampus [illustration adapted from Booker *et al.* (*56*)]. Created in BioRender. Li, Y. (2026) https://BioRender.com/d8t1ywf. Dual fluorescent in situ images of the hippocampus of WT mice showing *Chrm2* transcript (green) colocalizing with (**C**) pan-interneuron marker *Gad2* (magenta) but not with subtype-specific IN markers (**D**) *Sst* and (**E**) *Pvalb* (magenta). DAPI (4′,6-diamidino-2-phenylindole) stain showing the location of INs within the hippocampus. White arrows indicate *Chrm2-*positive neurons within the white bounding box in (C) to (E). (**F**) Immunofluorescent staining of Chrm2 (green) and MeCP2 (white) in *Gad2-Cre* mice injected with AAV-flex-tdTomato (red) to label INs. GAD2 colocalizes with CHRM2 and MeCP2 neurons indicated by (**G**) white arrow in bounding box from (F). (**H**) Upset plot showing DEG overlap patterns across IN subtypes. Venn diagram showing the largest number of shared DEGs between SST/PVALB^+^ INs and Chrm2^+^ INs (hypergeometric test *P* = 1.55 × 10^−32^). (**I**) The correlation plot showing Log_2_FC of the shared DEGs between Chrm2^+^ INs and SST/PVALB^+^ INs. (**J**) The GO comparison showing the biological processes enriched from shared versus unique DEGs of Chrm2^+^ INs and SST/PVALB^+^ INs.

In both of our single-nuclei datasets (male versus female and HET^NEG^ versus HET^POS^), we found four subtypes of INs (INs^Sst/Pvalb^, INs^Vip/Cck^, INs^Chrm2^, and INs^Nos1^). Of these, INs^Sst/Pvalb^ and INs^Chrm2^ showed the highest number of DEGs. Intriguingly, we observed that one specific IN population (INs^Chrm2^) exhibited substantially more snDEGs compared to other IN subtypes in both male and female (Figs. 2D and 4E). On the basis of markers that are expressed from these populations in our dataset and previous literature, we identified this highly affected population as trilaminar INs, characterized by high expression of cholinergic receptor muscarinic 2 (*Chrm2*) while lacking expression of somatostatin *(Sst*), parvalbumin (*Pvalb*), and other IN subtype markers (cluster 17 in Fig. 6A, left, and cluster 21 in Fig. 6A, right) (*54*–*57*). While previous studies have established that MeCP2 function is critical in somatostatin and parvalbumin expressing INs, with *Mecp2* KO in these subtypes alone causing RTT-like phenotypes (*58*), our findings identify an even more transcriptionally vulnerable population. Chrm2^+^ trilaminar INs exhibited three to four times more DEGs than SST/PVALB^+^ INs in both males and presymptomatic females (Fig. 2D), implicating trilaminar INs as another potentially important neuronal population in RTT pathogenesis.

Trilaminar neurons are long-range projection INs that span multiple hippocampal layers. As illustrated in the schematic (Fig. 6B), these cells have been previously described as located in the stratum oriens (SO) and stratum pyramidale (SP) and with their dendritic and axonal projections extending through stratum radiatum (SR) to reach the distal stratum lacunosum-moleculare (SLM) layer of the hippocampus, hence their designation as “trilaminar” (*56*). This strategic anatomical positioning places them at a key junction to modulate information flow across hippocampal layers.

To validate the identity and spatial location of trilaminar INs in the hippocampus, we used dual fluorescent in situ labeling of *Chrm2* with pan-interneuron marker *Gad2*, as well as two other major IN subtypes that also express *Chrm2* (*Sst* and *Pvalb*) (Fig. 6, C to E). We confirmed that *Chrm2* colocalizes with *Gad2* and labels a sparse number of INs located mainly within the SO and SP layers of CA1–CA3 (Fig. 6, C to E). We identified a subpopulation of *Chrm2* neurons that did not colocalize with *Sst* or *Pvalb* neurons and localized mostly in the CA1 subregion (Fig. 6, D and E). To better visualize the morphology of trilaminar INs, we labeled all hippocampal INs by injecting *Gad2-Cre* mice with an AAV-flex-tdTomato virus and stained tissue with antibodies against CHRM2 and MeCP2 (Fig. 6F). We found two morphologically distinct GAD2^+^/CHRM2^+^ populations that both colocalized with MeCP2: one located in the upper SO layers with horizontal projecting dendrites and another in or near the SP layers displaying perpendicular projecting dendrites that spanned the SO and SR hippocampal layers (Fig. 6G). This morphological heterogeneity within Chrm2^+^ trilaminar INs suggests potential functional diversity within the population.

### Chrm2^+^ trilaminar INs show amplified dysregulation along shared molecular pathways with SST/PVALB^+^ INs

To understand whether the extensive transcriptional changes in the Chrm2^+^ INs represent unique molecular mechanisms or exacerbated responses along shared pathways, we compared their DEGs with other IN subtypes. Overlapping the DEGs across IN clusters, we found that SST/PVALB^+^ INs overlapped the most with Chrm2^+^ INs DEGs, sharing 67 genes (hypergeometric test, *P* = 1.55 × 10^−32^), whereas 526 DEGs were unique to Chrm2^+^ INs and 190 were unique to SST/PVALB^+^ INs (Fig. 6H). This substantial molecular overlap is consistent with the anatomical connectivity between Chrm2^+^ trilaminar INs and SST^+^ OLM INs (*27*) in hippocampal circuits, suggesting coordinated dysfunction within this microcircuit. Despite sharing many of the dysregulated genes, Chrm2^+^ INs exhibited markedly larger transcriptional changes. Among 67 shared DEGs, 88.9% showed greater absolute log_2_ fold changes in Chrm2^+^ INs compared to SST/PVALB^+^ INs (Fig. 6I; paired Wilcoxon signed-rank test, *P* = 1.5 × 10^−10^). The strong directional correlation (Pearson *r* = 0.875) between fold changes in these populations indicates that Chrm2^+^ INs experience amplified dysregulation along similar molecular pathways rather than functionally distinct pathological mechanisms. Gene ontology analysis revealed that shared DEGs between SST/PVALB^+^ and Chrm2^+^ INs were enriched for core synaptic and neuron function pathways (Fig. 6J). Even genes that were uniquely differentially regulated in either Chrm2^+^ or SST/PVALB^+^ neurons showed enrichment in shared molecular pathways related to synaptic organization and activity (Fig. 6J).

Our snRNA-seq data revealed that trilaminar INs lacking MeCP2 (male NULL and HET^NEG^ samples) experience substantial overlap in transcriptional dysregulation (fig. S6A). The shared changes between NULL and HET^NEG^ trilaminar INs predominantly affect genes involved in neurotransmitter release and synaptic function, with most being down-regulated (fig. S6, B to D). In summary, these data suggest that trilaminar INs are particularly sensitive to cell autonomous loss of *Mecp2* compared to other hippocampal IN populations early in disease progression in both male and female RTT animal models.

## DISCUSSION

Like other X-linked disorders, RTT has sex-specific differences in clinical presentation due to XCI in females. This creates a mixture of cells expressing either a normal or mutant *MECP2* allele. How this mosaicism contributes molecularly to early RTT pathogenesis remains an open question. In our study, we investigate the mosaic (female) and nonmosaic (male) transcriptional changes in the hippocampus of the RTT mice, using both bulk and snRNA-seq approaches at early time points. Combining these techniques, we uncovered shared and distinct cell type–specific disease signatures across both male and female RTT models. We identified a set of early core RTT molecular changes common to both males and females, as well as early alterations of the transcriptome in a previously understudied inhibitory neuronal subtype.

### Early core molecular disease signature in the hippocampi of male and female RTT mice

Male and female RTT mice have notably different disease progression trajectories (*16*, *19*). Male *Mecp2^−/y^* mice have early onset of phenotypes, including learning and memory deficits, abnormal gait, irregular breathing, and hindlimb clasping beginning between 4 and 6 weeks, and die in young adulthood (~8 weeks). Female *Mecp2^+/−^* mice have normal survival and display progressive RTT-like neurological phenotypes as they age, typically appearing later in adulthood (~12 to 24 weeks) (*16*, *19*). Most studies therefore characterize male mice at a much earlier developmental time point than female mice. To exclude molecular changes due to age differences, we compared males and females at the same ages, using early time points in disease progression, 4 and 6.5 weeks. While most bulk transcriptional changes were unique between RTT models, with male RTT mice having ~10× more DEGs than female RTT mice, we found a core molecular disease signature consisting of 12 DEGs altered in the same direction at both time points and in both sexes. These core DEGs overlapped with DEGs from a recent study examining the transcriptional changes immediately occurring after the acute loss of MeCP2 in adult *Mecp2* conditional KO mice (*25*). This suggests that these genes are highly responsive to MeCP2 loss, and they presage disease phenotypes, underscoring their potential contribution to pathogenesis. Eleven of these DEGs were changed in the same direction across all datasets (*Mecp2*, *Pls3*, *Lpl*, *Gdf11*, *Cacna1g*, *Grin2d*, and *Htr1a* down-regulated and *Dlk1*, *Sdk1*, *Tpbg*, and *Efna5* up-regulated). The only exception is *Irak1*, which did not show a significant change upon loss of *Mecp2* in adult mice. Therefore, we propose that these changes are direct consequences of MeCP2 loss (*23*). Consistent with our data, most of the core DEGs (*Dlk1*, *Sdk1*, *Efna5*, *Pls3*, *Grin2d*, *Lpl*, and *Cacna1g*) are also dysregulated in the same direction in the bulk nuclei RNA-seq data from a study using 6-week-old male mice harboring common RTT missense mutations [Thr158 to Met(T158M) and Arg106 to Trp(R106W)] (*24*, *25*). Moreover, when examining previously published human cortical data (*23*), we found that 5 of the 12 core DEGs (*EFNA5*, *GDF11*, *HTR1A*, *CACNA1G*, and *MECP2*) were also significantly changed in the same direction. Several of these core RTT genes are associated with human neurodevelopmental disorders that overlap with RTT phenotypes. For example, mutations in both the NMDAR subunit *GRIN2D* and calcium voltage–gated channel subunit alpha 1G (*CACNA1G*) have been shown to cause developmental epileptic encephalopathies (*59*, *60*). The X-linked gene plastin 3 (*PLS3*) has been implicated in severe childhood-onset osteoporosis and scoliosis (*61*, *62*). Together, these findings not only show a core molecular RTT signature across several mouse models but highlight transcriptional changes that precede functional and behavioral changes.

Our results differ from a recent snRNA-seq study that examined the molecular changes over disease progression in cortical tissue from both male and female RTT mice harboring a point mutation at the 5′ transcriptional start site of *Mecp2* (*Mecp2-e1*), which ablates only one *Mecp2* isoform (*22*). These authors did not find the core RTT signature we found shared between male and female RTT models. Furthermore, they found that peak transcriptional changes occurred in presymptomatic female RTT mice, whereas changes in male RTT mice peaked in symptomatic stages of the disease. Because the adult KO study showed that transcriptomic changes preceded symptom onset and correlated with data from a constitutive KO male mouse model (*R* ~0.75) (*25*), we believe that the different results are in part driven by the use of the *Mecp2-e1* model where the *Mecp2-e2* isoform is still present and might compensate (*22*).

### Cell type–specific molecular changes in mosaic female RTT hippocampus

In contrast to bulk RNA-seq, we detected robust transcriptional changes in both female and male RTT samples at the single-nucleus level. The shared transcriptional changes in both male and female nuclei were positively correlated with bulk RNA-seq DEGs of *Mecp2^−/y^* mice, suggesting that molecular disease signatures in female RTT tissue (except for the core genes) are largely masked in bulk sequencing due to mosaicism, cellular heterogeneity, and differences in magnitude of gene expression changes. The snRNA-seq data allowed us to uncover cell type–specific changes that are either shared between or unique to male and female RTT models. Most of the shared transcriptional changes between males and females correlated with each other across cell types, except for oligodendrocytes, where overlapping DEGs lacked a consistent direction of changes between sexes. Previous research has shown that deletion of *Mecp2* specifically in oligodendrocytes did not cause overt RTT phenotypes (*44*) and restoring *Mecp2* in the oligodendrocyte lineage results in a minimal rescue. Together, these data indicate that the changes in oligodendrocyte transcription are likely secondary and influenced by the dysfunction of neighboring cells.

### Non–cell autonomous changes in hippocampal excitatory neurons reveal their responsiveness to the mosaic RTT environment

By sorting MeCP2^−^ and MeCP2^+^ nuclei for snRNA-seq, we were able to capture cell type–specific direct (cell-autonomous) and indirect (non–cell autonomous) transcriptional changes in the mosaic *Mecp2^+/−^* hippocampus. Unexpectedly, non–cell autonomous transcriptional changes were only detected in the MeCP2^+^ excitatory neuronal clusters of the hippocampus. These results add a unique layer of complexity to the effects of E/I imbalance in the female RTT model compared to the male and could explain some of the sex-specific differences observed in *Mecp2* rescue experiments (*42*, *43*). Prior studies have shown that deletion of *Mecp2* in the central nervous system (CNS) can recapitulate RTT phenotypes seen in germline deletion models (*17*). Consistent with that, rescue experiments restoring expression of *Mecp2* in the CNS can reverse most neurological phenotypes (*63*). However, notable differences have been observed between male versus female RTT models in cell type–specific rescue experiments. In female RTT mice, expressing *Mecp2* only in glutamatergic (Vglut2^+^) neurons completely rescued behavioral phenotypes, whereas expressing *Mecp2* only in GABAergic (Viaat^+^) neurons led to only partial improvements (*42*, *43*). In contrast, male mice showed similar improvement in survival and neurological phenotypes when *Mecp2* was in either glutamatergic or GABAergic neurons (*42*, *43*). Considering our molecular findings that MeCP2^+^ excitatory neurons in the mosaic RTT hippocampus are sensitive to the changes from neighboring MeCP2^−^ neurons, the more effective rescue observed in glutamatergic neurons could be explained by rescuing a larger proportion of dysfunctional neurons early in female RTT mice (fig. S5C). Glutamatergic neurons, particularly in the DG, might also be more responsive to the environmental signals or changes in network activity since this region is a niche for neurogenesis in the adult brain and is primed to respond to external cues (*64*).

Our findings align with previous studies that have found non–cell autonomous transcriptional changes in MeCP2^+^ cells of RTT female cortical neurons (*22*–*24*). Work from Sharifi *et al.* (*22*) showed large non–cell autonomous changes in both E/I neurons in the cortex at the earliest time point (P30), which by P60 were largely restricted to glutamatergic neurons. Renthal *et al.* (*23*) focused their analysis of non–cell autonomous changes exclusively in cortical glutamatergic neurons and found non–cell autonomous changes in the 12- to 20-week-old females RTT mice. Johnson *et al.* (*24*) found non–cell autonomous changes in MeCP2^+^ cells in female mice modeling common RTT-causing mutations (T158M and R106W); however, they did not examine cell type specificity further. Our study focuses on both E/I neurons and reveals regional cell type–specific differences, with a non–cell autonomous influence particularly on excitatory neurons of the hippocampus early in disease pathogenesis. Consistent with our transcriptional findings, previous studies examining morphological changes of MeCP2^+^ cells in older (5 to 21 months) female *Mecp2^+/−^* mice compared to control neurons found significant decreases in soma size, nuclear size, and spine density (*48*, *65*). Although functional characterization of basal electrophysiological properties of MeCP2^+^ neurons in *Mecp2^+/−^* mice have not found clear differences compared to controls (*26*, *27*), our findings suggest that mosaicism within the female hippocampus influences the transcriptome of glutamatergic excitatory neurons early in the disease process and could influence functional deficits via both direct and indirect molecular mechanisms.

### Technical consideration in MeCP2-sorted single-nucleus analysis

Our antibody-based FANS strategy successfully separated MeCP2^+^ and MeCP2^−^ populations, although the approach resulted in differential cell type capture rates between neurons and glia. Specifically, because of the higher MeCP2 abundance in neurons compared to glia, the HET-NEG fraction captured proportionally more glial cells. To address this technical artifact and ensure robust statistical comparisons, we focused our differential expression analysis on neuronal populations and more specifically on the abundant neuronal clusters (DG, CA1, and CA3 regions) that have comparable cell numbers across experimental groups. While this conservative approach may increase false-negative rates for rare cell types in the MeCP2-sorted dataset, our identification of trilaminar IN vulnerability is not subject to this limitation as these findings were independently replicated across three separate comparisons (male NULL versus WT, female HET versus WT, and HET-NEG versus WT), demonstrating biological robustness rather than sampling artifacts.

### Transcriptional changes are enriched in trilaminar INs across RTT models

Our snRNA-seq studies uncovered a particular subcluster of INs that displayed ~three to four times more significant transcriptional changes upon loss of *Mecp2* compared to other IN populations. The molecular changes in this IN subcluster were notable given that they were found across three distinct comparisons in both male and female RTT and are unique to MeCP2^−^ nuclei of female RTT samples. These INs have molecular characteristics of hippocampal trilaminar INs, including high expression of *Chrm2* while lacking expression of *Sst* and *Pvalb* (*54*), consistent with a previous study that profiled different subclasses of CA1 inhibitory neurons (*66*). Although trilaminar hippocampal INs have not been extensively characterized, studies have shown that they localize to the SO and pyramidale layers and they input onto somatostatin INs in the OLM (SST^+^ OLM) (*54*–*57*, *66*, *67*). This connection is particularly intriguing because previous research has shown that MeCP2-deficient SOM^+^ OLM INs in *Mecp2^+/−^* females are weakly recruited into an associative memory ensemble, resulting in larger CA1-excitatory neuronal engrams and deficits in long-term memory recall (*27*). Moreover, *Chrm2* KO mice have significant deficits in memory and synaptic plasticity (*68*), suggesting that disruption of cholinergic signaling in these neurons may contribute to cognitive dysfunction. The disproportionate vulnerability of these neurons to MeCP2 loss, given the extensive transcriptional dysregulation of genes involved in neurotransmission and synaptic function, highlights a potentially important mechanism underlying early RTT pathogenesis. It will be crucial to investigate how this specific neuronal population contributes to circuit dysfunction in RTT and whether therapeutically targeting these neurons could ameliorate cognitive symptoms of the disorder.

In summary, our study revealed the following: (i) A core set of genes consistently dysregulated across different time points in both male and female RTT mouse models, indicating shared and early molecular mechanisms in RTT pathogenesis; (ii) snRNA-seq uncovered cell–type–specific transcriptional alterations that were masked in bulk tissue, revealing a disease signature in female RTT mice that was obscured by bulk RNA-seq and highlighting the importance of cellular resolution in studying mosaic disorders; (iii) notable cell type–specific differences between male and female models despite many shared gene expression changes; (iv) distinct non–cell autonomous effects primarily affecting excitatory neurons in the mosaic female RTT hippocampus; and (v) extensive transcriptional changes unique to the MeCP2 negative trilaminar neurons in both male and female RTT mice. Together, these data reveal how female mosaicism influences distinct molecular pathways in the hippocampus, providing crucial insights that might extend beyond RTT to other X-linked neurodevelopmental disorders such as DDX3X syndrome and CDKL5 deficiency disorder. These findings underscore the fundamental importance of considering sex-specific molecular mechanisms in disease pathogenesis and highlight the critical need for therapeutic strategies that account for the unique biology of female mosaicism across X-linked neurological conditions.

## MATERIALS AND METHODS

### Animals

Mice were maintained on a C57BL/6 background on a 14-hour:10-hour light:dark cycle at 68° to 72°F and 30 to 70% humidity and fed standard mouse chow and water ad libitum*.* Up to five mice were housed per cage. *Mecp2^−/y^* and *Mecp2^+/−^* mice were maintained by crossing *Mecp2^+/−^* females to WT C57BL/6J males (*16*). Experimental mice were obtained through crossing *Mecp2^+/−^* to C57BL/6J from the Jackson Laboratory. Homozygous male and female Gad2-IRES-Cre knock-in C57BL/6J mice [*Gad2^tm2(cre)Zjh^*] were obtained from Jax (strain #028867), and homozygous mice were bred together and genotyped according to the Jax protocol. The Baylor College of Medicine Institutional Animal Care and Use Committee approved all research and animal care procedures (protocol AN-1013).

### Bulk RNA-seq

Hippocampi were dissected from P28 or P45 mice and flash-frozen in liquid nitrogen (*n* = 4 per genotype). Tissues were homogenized using a TissueLyser II (Qiagen, #85300) with a customized program (30.0 Hz, 30 s × 2). Total RNA was extracted immediately using the RNeasy Mini Kit (Qiagen, #75106) according to the manufacturer’s instructions, including on-column deoxyribonuclease I treatment (Qiagen, #79254) to remove genomic DNA. RNA quality was assessed by Genewiz using TapeStation and Qubit, followed by library preparation and sequencing with ribosomal RNA depletion method for mRNA with unique molecular identifier (UMI) controls. Libraries were sequenced at ~50 million 150–base pair paired-end reads per sample for transcriptomic profiling. STAR v2.7.9a was used to align trimmed reads to the *Mus musculus* genome (mouse_mm10v23 and gencode.vM23.primary_assembly.annotation.gtf for annotation) to obtain read counts with default parameters (*69*). Default STAR parameters were used other than –sjdbOverhang 149. The alignment rates for all samples were more than 80%. The read count matrix was used as input for differential gene expression analysis with DESeq2 (v1.40.2) (*70*). Genes with a mean count <10 were filtered out to remove lowly expressed genes. DEGs were identified using an adjusted *P* value threshold of <0.05 and abs(log_2_FC) > 0.15.

Overlap of the 12 core RTT DEGs with acute KO data was done using DEGs from the 6 weeks post–MeCP2 deletion (*25*). A hypergeometric test was performed using 1072 DEGs in adult KO and 20,000 for background genes, and observed overlap was 11 genes.

### CIBERSORTx deconvolution

After excluding mitochondrial genes and genes involved in cell cycle, ribosome biogenesis, and apoptosis (GO:0007049, GO:0042254, and GO:0008637), we selected the top 300 genes for each cell type ranked by log_2_ fold change using the FindAllMarkers function in the Seurat package (v5.4.0). For each sample and cell type, 50 cells were randomly selected, and their expression profiles were used to generate a gene expression matrix, which was subsequently used by CIBERSORTx (*71*) to build the single-cell signature matrix. In parallel, counts per million values for bulk RNA-seq samples were computed using edgeR (v4.0.16) (*72*).

The Input Cell Expression module in CIBERSORTx, with the high-resolution option enabled, was then applied to estimate cell type proportions and infer cell type–specific expression profiles from bulk RNA-seq data. A gene subset file consisting of the union of bulk RNA-seq DEGs and the top 180 highly variable genes was used for gene expression imputation (*n* = 1250). The Wilcoxon test was used to compute the *P* value of the imputed gene expression between sample groups (Null_M versus WT_M and Het_F versus WT_M). The cell type expression profiles could not be inferred for OPC and microglia due to insufficient statistical power.

### Tissue collection and nuclei isolation

Animals were decapitated after anesthetization with 3% isoflurane. The hippocampi of mice were dissected on ice, flash frozen in liquid nitrogen, and stored at −80°C until further use. The nuclei isolation protocol was adapted from 10x Genomics for adult brain tissue. Briefly, on the day of nuclei isolation, frozen hippocampi were directly homogenized in ice-cold lysis buffer [10 mM tris-HCl (pH 7.4), 10 mM NaCl, 3 mM MgCl_2_, and 0.1% IGEPAL CA-630 from Sigma-Aldrich] and incubated on ice for 10 min. The tissue lysate was passed through a 30-μm MACS SmartStrainer filter to remove larger debris and then centrifuged at 500*g* for 5 min at 4°C. The supernatant was removed, and the nuclei pellet was washed with wash buffer [1× Dulbecco’s phosphate-buffered saline (DPBS) with 1% bovine serum albumin (BSA, Thermo Fisher Scientific, #37525)] and then centrifuged at 500*g* for 5 min at 4°C. A total of two washes were performed. For samples requiring antibody staining, MeCP2-conjugated phycoerythrin antibody (Cell Signaling Technology #34113S) was used and incubated at 1:1000 dilution at 4°C for 30 min followed by two washes. The final nuclei suspension was checked under Thermo Fisher Scientific Countless II using Trypan blue to ensure the particle concentration was below 1 × 10^7^ to avoid clogging on the SONY SH800s machine.

### Nuclei sorting

Nuclei suspensions were stained with DRAQ5 fluorescent probe (1:1000 dilution) for 5 min before sorting. The forward scatter (FSC) threshold was set at 1.50% with an FSC gain of 11, and the back scatter (BSC) gain was adjusted to 30.5%. Nuclei gates were first determined by passing samples through a gate (BSC-A versus FSC-A) to filter out debris using control nuclei without DRAQ5 staining and then through two single-nucleus gates (FSC-H versus FSC-A and BSC-H versus BSC-W). For experiments separating HET^POS^ and HET^NEG^ groups, the HET^NEG^ gate was determined using stained nuclei from NULL male. Before sorting, the 1.5-ml collection tube was coated with 1% BSA and filled with 50 μl of single-cell sorting buffer (1× DPBS with 1% BSA). A total of 100,000 to 150,000 nuclei were collected for each sample. For HET-sorted samples, HET^POS^ and HET^NEG^ group were sorted into separate tubes. After sorting, nuclei were spun down at 1500*g* for 10 min at 4°C to recover all nuclei and then resuspended to roughly a concentration of 1000 nuclei/μl. A total of 10,000 to 12,000 nuclei were loaded on the 10X Chip.

### Library construction and sequencing

The cDNA libraries were constructed by 10x Genomics 3′ v3.1 or 5′ HT Reagent Kits v2 (Dual index) following the user’s guide. Briefly, a total volume of 77.4 μl of nuclei suspension (10,000 nuclei targeted) was mixed with reverse transcription master mix before loading into Chromium Chip G/N. Droplets containing nuclei, reverse transcription reagents, and barcoded gel beads were generated by Chromium X Controller. The first-strand cDNA was then amplified and checked using TapeStation with HS D5000 ScreenTape to assess cDNA amplification. Then, the cDNA libraries were fragmented, size selected using SPRI beads, and ligated with sequencing adapters and sample indices. The cDNA libraries were sequenced by Illumina NovaSeq 6000 using S4 flow cell chemistry.

### Data preprocessing

The reads were aligned to 10X Genomics mouse reference genome mm10 (2020-A). The alignment and quantification of UMIs were performed on 10X Genomics Cloud by the Cell Ranger pipeline v7.0.1 with default parameters including intronic reads mapping.

We performed quality control for the snRNA-seq data using the R package Seurat (v5.0.0). Specifically, we used the following criteria to identify low-quality nuclei: (i) UMI counts <500 or UMI counts >30,000; (ii) number of genes <500 or number of genes >6000; and (iii) mitochondrial gene expression ratio >2%. We filtered out all low-quality nuclei. For doublet removal, we used the R package scDblFinder (v1.12.0) (*73*) to detect potential doublets and removed all nuclei classified as doublets. Nuclei from different samples were integrated with batch effect correction using the R package harmony (v1.2.0) (*74*). The integrated data were analyzed following the Seurat workflow. Specifically, we (i) normalized the raw counts (normalization.method = “LogNormalize”), (ii) selected the top 2000 highly variable genes and scaled the data based on these highly variable genes, (iii) used the selected highly variable genes to calculate the first 50 principal components, (iv) clustered nuclei using the FindNeighbors function (dims = 1:20) and the FindClusters function (resolution = 0.5), and (v) reduced the dimension of the data through Uniform Manifold Approximation and Projection (dims = 1:20).

### Cell annotation

The top marker genes of different clusters were identified using the FindAllMarkers function (only.pos = TRUE, min.pct = 0.25, logfc.threshold = 0.25) from the R package Seurat (v5.0.0). Cell type identity was determined by comparing these top marker genes to published reference datasets (*46*, *47*, *54*, *66*, *75*, *76*). Cell type composition testing was performed using propeller function from R package speckle (*77*), taking into account individual samples within each experimental group.

### Identification of DEGs in snRNA-seq

The DEG analysis was performed using the Seurat function FindMarkers(min.pct = 0.1) with the MAST method (v1.24.1) (*78*). With the output from FindMarkers, DEGs were defined using the following criteria: (i) the absolute value of average log_2_ fold change >0.15; (ii) the adjusted *P* value <0.05. Upset plots for DEG numbers among different comparisons were generated using the R package ComplexUpset (v1.3.5). Unique snDEGs were defined as genes that were differentially expressed in one or more clusters, regardless of the number of clusters in which they appeared. A WT-to-WT comparison analysis was performed (results in table S7) to determine the false-positive rate (<0.04%) in both datasets to evaluate our cutoff decision across cluster for significant DEGs. This analysis used an identical analytical pipeline to our experimental comparisons. Our experimental design included a balanced genotype distribution across sequencing batches, with both mutant and WT samples from both sexes represented in each batch. This balanced design ensures that any batch effects are equally distributed across genotypes and would not systematically drive DEGs in genotype comparisons; consequently, the WT-versus-WT analysis provides a meaningful estimate of the false positive background under equivalent analytical conditions.

### Bootstrap consensus pseudobulk analysis

For each biological sample (or within a given cell type), we randomly partitioned nuclei into subsamples of 500 cells each, creating independent pseudo-replicates while preserving biological replicate structure. This subsampling process was repeated across 10 independent iterations using different random seeds. Within each iteration, we aggregated raw UMI counts across nuclei to generate pseudobulk expression profiles and then performed differential expression analysis using DESeq2 (*70*) with the original biological sample identity as a batch covariate (design = ~batch + group). Consensus DEGs were defined as those detected as significant (adjusted *P* value <0.05, |log^2^ fold change| >0.15) in at least 5 of 10 iterations. For cross-platform validation, we compared consensus DEGs against bulk RNA-seq data from matching genotypes using hypergeometric tests for enrichment, Pearson correlation for fold change concordance, and calculated directional concordance as the percentage of overlapping genes showing the same direction of change.

### Hypergeometric enrichment testing

To assess whether overlaps between DEG sets exceeded random expectation, we performed hypergeometric tests using the phyper function in R. For each comparison, the universe size was defined as the total number of genes tested in both datasets, calculated as the minimum of the total genes detected in each study. The hypergeometric test parameters were as follows: q = observed overlap −1, m = number of DEGs in the first gene set, n = universe size - m (genes not in the first set), and k = number of DEGs in the second gene set, with lower.tail = FALSE to calculate upper-tail probabilities. Expected overlap by chance was calculated as (m × k)/universe, and fold enrichment was computed as the ratio of observed to expected overlap. Statistical significance was determined at α = 0.05. To assess directional concordance, we additionally performed separate hypergeometric tests for up-regulated and down-regulated gene subsets within each cluster, using only up-regulated or down-regulated DEGs from each dataset as input, respectively. For overlapping genes, we assessed directional concordance by calculating the percentage of genes showing the same sign of log_2_ fold change across datasets. Correlation between datasets was evaluated using Pearson correlation tests on log_2_ fold changes of overlapping genes. All correlation tests and *P* values were computed using R function (cor.test).

### Pathway analysis

GO pathway enrichment analysis was performed using the R package clusterProfiler (*79*) (v4.6.2) with the function enrichGO (OrgDb = org.Mm.eg.db (v3.16.0), pAdjustMethod = “BH,” pvalueCutoff = 0.05, qvalueCutoff = 0.05). The up-regulated (log_2_FoldChange >0) and down-regulated (log_2_FoldChange <0) DEGs were used as input to enrich up-regulated and down-regulated pathways.

### Trilaminar IN labeling

P0 pups from homozygous *Gad2^tm2(cre)Zjh^* breeding received bilateral intracerebroventricular injections with AAV9-FLEX-tdTomato virus obtained from Addgene (catalog #28306-AAV9), using a 1-μl volume with 1.5 × 10^10^ viral particles per hemisphere. Mice were harvested 25 days later for downstream processing. Following viral injection, mice at age P25 were completely anesthetized by giving an intraperitoneal injection of Rodent Combo III (1.5 ml/kg) and then perfused with 30 ml of cold 1× PBS and 30 ml of 4% paraformaldehyde (PFA). The brains were extracted and incubated in 4% PFA overnight. The brains were washed with cold 1× PBS and then incubated in 30% sucrose with 0.01% sodium azide solution for at least 48 hours until sectioning. A Leica SM2010R sliding microtome at −25°C stage temperature was used to section the right hemisphere along the sagittal plane at 60-μm thickness. Free floating serial sections were stored in 1× PBS + 0.01% sodium azide at 4°C until further processing. Sections were washed three times with PBS and incubated in permeabilization buffer (0.3% Triton X-100 in PBS) for 5 min at room temperature. Sections were placed in blocking buffer (0.3% Triton X-100, 3% normal donkey serum) for 30 min at room temperature and then incubated with primary antibodies (rtChrm2 MAB367 1:500, rbMeCP2 CST34113S 1:500) with 1% BSA in blocking buffer overnight, rocking at 4°C. Sections were washed three times with PBS and then incubated with secondary antibodies at 1:1000 dilutions (donkey anti-rabbit 647 from Jackson ImmunoResearch 711-605-152 and goat anti-rat 488 from Thermo Fisher Scientific A-11006) in blocking buffer with 1% BSA for 1 hour at room temperature. After final washes with PBS, the sections were incubated for 10 min in 4′,6-diamidino-2-phenylindole (5 μg/ml) solution and then mounted onto slides and allowed to dry. Coverslips were mounted using Prolong Gold mounting media, and slides were allowed to dry overnight. Stained sections were imaged on a Leica SP8X confocal microscope using 20× oil objective.

### Dual-color fluorescence RNA in situ hybridization

Fluorescent in situ hybridization was performed by the RNA In Situ Hybridization Core at BCM using previously described methods (*80*). Briefly, fresh frozen adult mouse tissue was used and sectioned along the sagittal plane at 25 μm. Digoxigenin (DIG)-labeled, or fluorescein [fluorescein isothiocyanate (FITC)]–labeled mRNA antisense probes were generated for *Gad2*, *Chrm2*, *Sst*, and *Pvalb* from Allen Brain Atlas sequences (https://mouse.brain-map.org). The probe templates were generated by polymerase chain reaction using reverse-transcribed mouse cDNA as template and DIG or FITC RNA labeling kits from Roche (Sigma-Aldrich). Slides were imaged on a Leica SP8X confocal microscope using 20× objective.
