# Supplementary material for: Single-nucleus profiling reveals a core disease signature and cell type–specific vulnerabilities in early Rett syndrome
Source: Sci Adv. 2026 Jun 10;12(24):eaeb4265. doi: 10.1126/sciadv.aeb4265 (PMC13251834; doi:10.1126/sciadv.aeb4265)
Supplement: Supplementary file 1 — Figs. S1 to S6 Legends for tables S1 to S8 [file sciadv.aeb4265_sm.pdf]

Science Advances
AAAS

Supplementary Materials for

Single-nucleus profiling reveals a core disease signature and cell type–specific vulnerabilities in early Rett syndrome

Yan Li *et al.*

Corresponding author: Ashley G. Anderson, ashley.anderson3@bcm.edu; Huda Y. Zoghbi, hzoghbi@bcm.edu

Sci. Adv. **12**, eaeb4265 (2026)
DOI: 10.1126/sciadv.aeb4265

The PDF file includes:

Figs. S1 to S6
Legends for tables S1 to S8

Other Supplementary Material for this manuscript includes the following:

Tables S1 to S8

Supplemental Figure 1

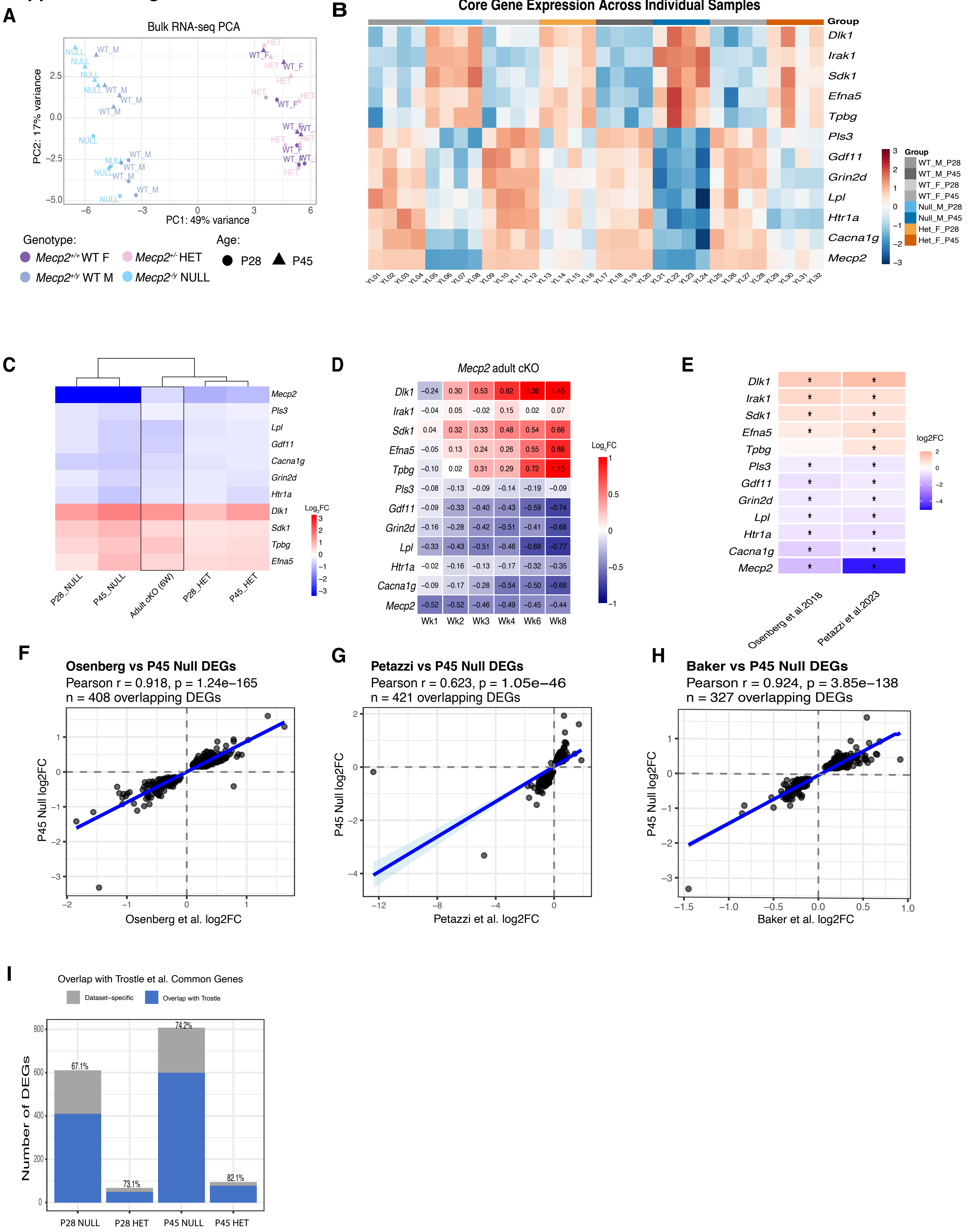

Fig. S1. PCA of bulk RNA-seq samples and cross comparison with other *Mecp2* gene expression data. (**A**) Principal component analysis (PCA) of all 32 hippocampal samples from 2male and female WT, NULL, and HET samples at two timepoints. (**B**) Heatmap of z-scored log2 normalized counts of 12 core DEGs expression across individual samples. (**C**) Heatmap comparing the log2FC of 12 core DEGs found in the hippocampal adult *Mecp2* cKO bulk RNA-seq to our dataset. (**D**) Heatmap of log2FC of all core DEGs across the time course of adult *Mecp2* cKO bulk RNA-seq at week (wk) 1, 2, 3, 4, 6, and 8 post-deletion of *Mecp2*. (**E**) Heatmap comparing the log2FC of 12 core DEGs found in previously published hippocampal data sets. An * represents significant. (**F-H**) Correlation of log2FC between our P45 Null DEGs and published hippocampal datasets. (**I**) Overlap between DEGs from our dataset and Trostle et a. 2023 meta-analysis common core disease signatures. Blue: overlap with Trostle; gray: dataset specific.

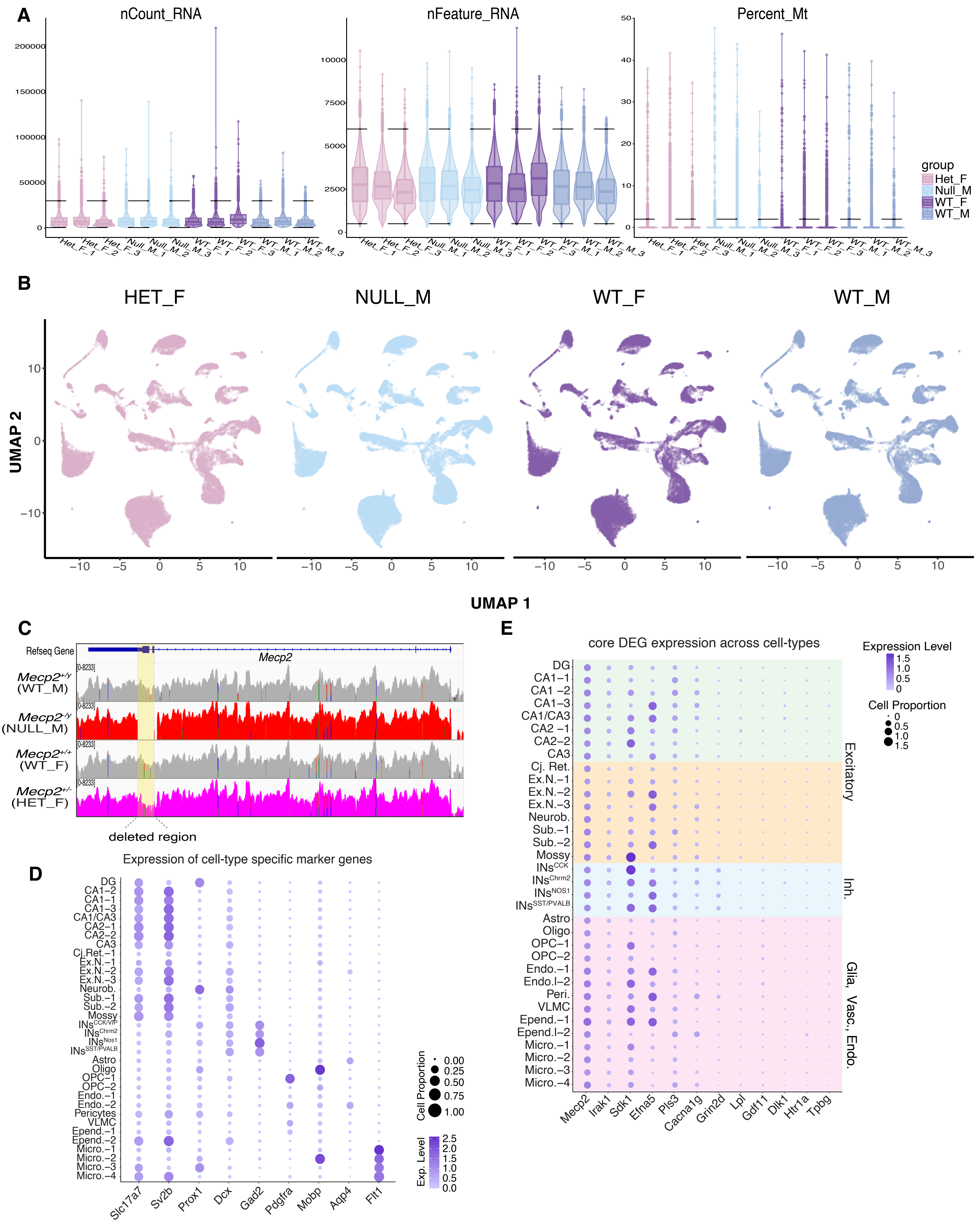

A
nCount_RNA
nFeature_RNA
Percent_Mt
group
Het_F
Null_M
WT_F
WT_M
Het_F_1 Het_F_2 Het_F_3 Null_M_1 Null_M_2 Null_M_3 WT_F_1 WT_F_2 WT_F_3 WT_M_1 WT_M_2 WT_M_3
B
HET_F
NULL_M
WT_F
WT_M
UMAP 2
UMAP 1
C
Refseq Gene
Mecp2
Mecp2+/y (WT_M)
Mecp2-/y (NULL_M)
Mecp2+/+ (WT_F)
Mecp2+/- (HET_F)
[0-8233]
deleted region
D
Expression of cell-type specific marker genes
DG
CA1−2
CA1−1
CA1−3
CA1/CA3
CA2−1
CA2−2
CA3
Cj.Ret.−1
Ex.N.−1
Ex.N.−2
Ex.N.−3
Neurob.
Sub.−1
Sub.−2
Mossy
INsCCK/VIP
INsChrm2
INsNos1
INsSST/PVALB
Astro
Oligo
OPC−1
OPC−2
Endo.−1
Endo.−2
Pericytes
VLMC
Epend.−1
Epend.−2
Micro.−1
Micro.−2
Micro.−3
Micro.−4
Slc17a7 Sv2b Prox1 Dcx Gad2 Pdgfra Mobp Aqp4 Flt1
Cell Proportion
0.00
0.25
0.50
0.75
1.00
Exp. Level
2.5
2.0
1.5
1.0
0.5
0.0
E
core DEG expression across cell-types
Expression Level
Cell Proportion
Excitatory
Inh.
Glia, Vasc., Endo.
DG
CA1−1
CA1 −2
CA1−3
CA1/CA3
CA2 −1
CA2−2
CA3
Cj. Ret.
Ex.N.−1
Ex.N.−2
Ex.N.−3
Neurob.
Sub.−1
Sub.−2
Mossy
INsCCK
INsChrm2
INsNOS1
INsSST/PVALB
Astro
Oligo
OPC−1
OPC−2
Endo.−1
Endo.l−2
Peri.
VLMC
Epend.−1
Epend.l−2
Micro.−1
Micro.−2
Micro.−3
Micro.−4
Mecp2 Irak1 Sdk1 Efna5 Pls3 Cacna1g Grin2d Lpl Gdf11 Dlk1 Htr1a Tpbg

Fig. S2. snRNA-seq quality control measures across genotypes and cell type annotation of clusters. (**A**) Distribution of key quality control metrics across genotype groups. Violin plots show distribution of unique molecular identifiers (UMIs) (nCount_RNA), genes detected 4(nFeature_RNA), and percent mitochondria (Percent_Mt) with the black dotted lines identifying the cutoff quality control values (see methods). Following quality control filtering, there were no significant difference between genotype groups (Kruskal-Wallis test, p = 0.74). (**B**) UMAP showing nuclei labelled by genotype (**C**) Track files of all *Mecp2* reads from the single-nuclei dataset separated by genotype, showing that no reads map to the deleted exons 3 and 4 (yellow highlight) of *Mecp2*-/y mice and reads in the exons are reduced in *Mecp2*+/- mice. (**D**) Bubble plot showing expression of top cell type marker genes within each cluster. (**E**) Bubble plot showing expression of core DEGs across cell types.

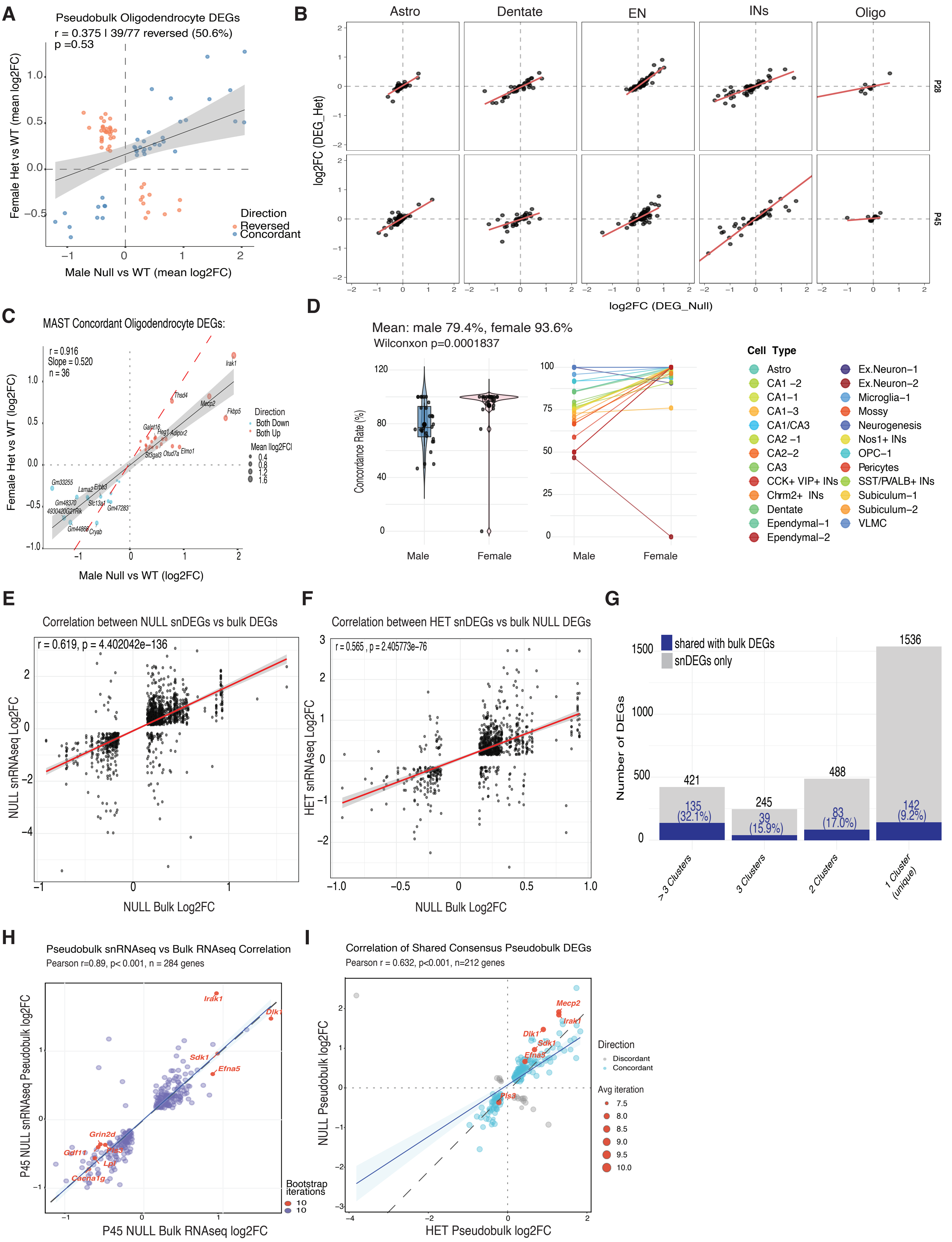
A
Pseudobulk Oligodendrocyte DEGs
r = 0.375 | 39/77 reversed (50.6%)
p =0.53
Female Het vs WT (mean log2FC)
Male Null vs WT (mean log2FC)
Direction
Reversed
Concordant
B
Astro
Dentate
EN
INs
Oligo
P28
P45
log2FC (DEG_Het)
log2FC (DEG_Null)
C
MAST Concordant Oligodendrocyte DEGs:
r = 0.916
Slope = 0.520
n = 36
Irak1
Thsd4
Mecp2
Fkbp5
Galnt16
Heg1
Adipor2
St3gal3
Otud7a
Elmo1
Gm33255
Lama2
Erbb3
Gm48370
Slc13a1
Gm47283
4930420G21Rik
Gm44868
Cryab
Direction
Both Down
Both Up
Mean |log2FC|
0.4
0.8
1.2
1.6
Female Het vs WT (log2FC)
Male Null vs WT (log2FC)
D
Mean: male 79.4%, female 93.6%
Wilconxon p=0.0001837
Concordance Rate (%)
Male
Female
Cell Type
Astro
CA1 −2
CA1−1
CA1−3
CA1/CA3
CA2 −1
CA2−2
CA3
CCK+ VIP+ INs
Chrm2+ INs
Dentate
Ependymal−1
Ependymal−2
Ex.Neuron−1
Ex.Neuron−2
Microglia−1
Mossy
Neurogenesis
Nos1+ INs
OPC−1
Pericytes
SST/PVALB+ INs
Subiculum−1
Subiculum−2
VLMC
E
Correlation between NULL snDEGs vs bulk DEGs
r = 0.619, p = 4.402042e−136
NULL snRNAseq Log2FC
NULL Bulk Log2FC
F
Correlation between HET snDEGs vs bulk NULL DEGs
r = 0.565 , p = 2.405773e−76
HET snRNAseq Log2FC
NULL Bulk Log2FC
G
shared with bulk DEGs
snDEGs only
Number of DEGs
421
135 (32.1%)
245
39 (15.9%)
488
83 (17.0%)
1536
142 (9.2%)
>3 Clusters
3 Clusters
2 Clusters
1 Cluster (unique)
H
Pseudobulk snRNAseq vs Bulk RNAseq Correlation
Pearson r=0.89, p< 0.001, n = 284 genes
Irak1
Dlk1
Sdk1
Efna5
Grin2d
Gdf11
Pls3
Lpl
Cacna1g
P45 NULL snRNAseq Pseudobulk log2FC
P45 NULL Bulk RNAseq log2FC
Bootstrap iterations
10
10
I
Correlation of Shared Consensus Pseudobulk DEGs
Pearson r = 0.632, p<0.001, n=212 genes
Mecp2
Irak1
Dlk1
Sdk1
Efna5
Pls3
Direction
Discordant
Concordant
Avg iteration
7.5
8.0
8.5
9.0
9.5
10.0
NULL Pseudobulk log2FC
HET Pseudobulk log2FC

Fig. S3. Cross-platform validation of transcriptional changes reveals strong concordance between bulk and single-nucleus RNA-seq. (**A**) Pseudobulk aggregation of oligodendrocyte snDEGs shows strong correlation with male NULL bulk DEGs Reversed genes represent 50.6% 6of all overlapped genes (**B**)Deconvolution of bulk RNA-seq. Correlation between Log2FC in HET vs NULL in astrocyte, Dentate (P28: $r=0.85$, $p<0.001$; P45: $r=0.60$, $p<0.001$), excitatory neurons (EN) (P28: $r=0.91$, $p<0.001$; P45: $r=0.77$, $p<0.001$), inhibitory neurons (INs) (P28: $r=0.84$, $p<0.001$; P45: $r=0.94$, $p<0.001$), and Oligodendrocytes (Oligo) (P28: $r=0.89$, $p<0.001$; P45: $r=0.27$, $p=0.301$) (**C**) Correlation of the 36 concordance DEGs from MAST analysis (**D**) The male/female concordance analysis between DEGs identified in Oligodendrocyte and other cell types (Left) and parallel coordinate plot tracking individual cell type concordance between male and female samples. Each line represents one cell type colored by cell type. Correlation plots of log2FC between male NULL bulk RNA-seq with snDEGs from (**E**) male and (**F**) female. (**G**) Bar plot showing the number of male snDEGs that are unique to one cell type (1 cluster) or shared across multiple clusters (gray bars), with blue bars indicating overlap with male bulk DEGs (P45) and percentages showing overlap rates. Data from male snDEGs. (**H**) Pseudobulk snRNAseq vs bulk RNA-seq correlation for P45 NULL samples. Core genes are labeled in red. (**I**) Correlation between male and female pseudobulk analysis. Point size represents average number of bootstrap iterations (out of 10) for each gene. Core RTT genes are labeled in RTT.

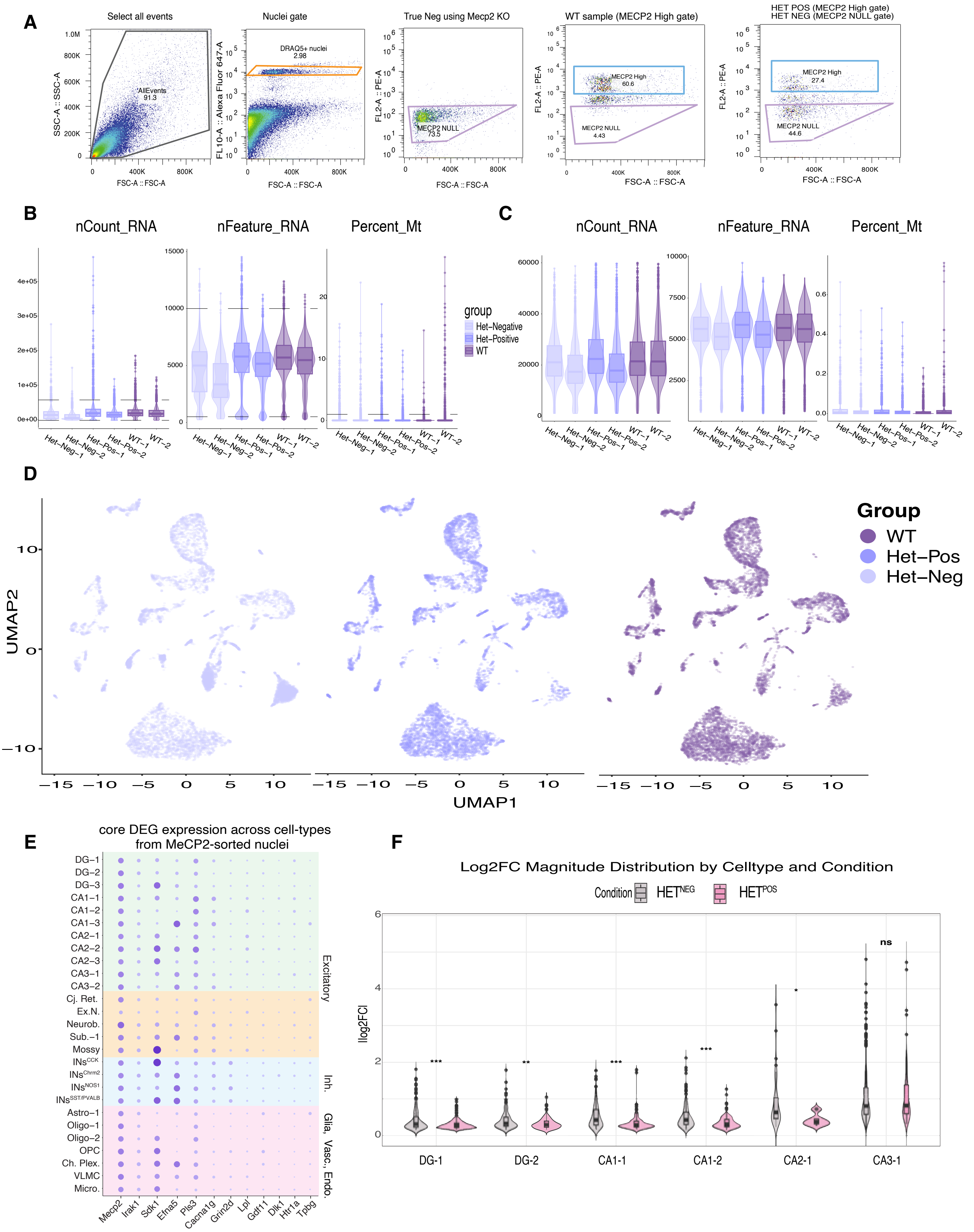
A
Select all events
Nuclei gate
True Neg using Mecp2 KO
WT sample (MECP2 High gate)
HET POS (MECP2 High gate)
HET NEG (MECP2 NULL gate)
AllEvents 91.3
DRAQ5+ nuclei 2.98
MECP2 NULL 73.5
MECP2 High 60.6
MECP2 NULL 4.43
MECP2 High 27.4
MECP2 NULL 44.6
SSC-A :: SSC-A
FSC-A :: FSC-A
FL10-A :: Alexa Fluor 647-A
FL2-A :: PE-A
B
C
nCount_RNA
nFeature_RNA
Percent_Mt
group
Het-Negative
Het-Positive
WT
Het-Neg-1
Het-Neg-2
Het-Pos-1
Het-Pos-2
WT-1
WT-2
D
Group
WT
Het-Pos
Het-Neg
UMAP1
UMAP2
E
core DEG expression across cell-types from MeCP2-sorted nuclei
DG-1
DG-2
DG-3
CA1-1
CA1-2
CA1-3
CA2-1
CA2-2
CA2-3
CA3-1
CA3-2
Cj. Ret.
Ex.N.
Neurob.
Sub.-1
Mossy
INsCCK
INsChrm2
INsNOS1
INsSST/PVALB
Astro-1
Oligo-1
Oligo-2
OPC
Ch. Plex.
VLMC
Micro.
Excitatory
Inh.
Glia, Vasc., Endo.
Mecp2
Irak1
Sdk1
Efna5
Pls3
Cacna1g
Grin2d
Lpl
Gdf11
Dlk1
Htr1a
Tpbg
F
Log2FC Magnitude Distribution by Celltype and Condition
Condition
HETNEG
HETPOS
|log2FC|
***
**
***
***
*
ns
DG-1
DG-2
CA1-1
CA1-2
CA2-1
CA3-1

Supplemental Figure 4. snRNA-seq quality control measures within the hippocampus of female RTT mice. **A**) FANS gating strategy for isolation of MeCP2+ and MeCP2- nuclei from female hippocampus. **B**) Violin plots show distribution of unique molecular identifiers (UMIs) (nCount_RNA), genes detected (nFeature_RNA), and percent mitochondria (Percent_Mt) with the black dotted lines identifying the cutoff quality control values and **C)** same QC plot post-filtering for neuronal population. Following filtering, nCount_RNA shows no significant difference between genotype groups (Kruskal-Wallis test, $p = 0.18$). **D)** UMAP showing nuclei labelled by sample groups. **E**) Bubble plot showing expression of core DEGs across cell types. **F)** Violin plots showing the distribution of absolute log2 fold change (|log2FC|) values for snDEGs in HET^{NEG} (MeCP2-, gray) and HET^{POS} (MeCP2+, pink) neurons compared to WT controls across different hippocampal neuronal subtypes. Each point represents an individual snDEG. HET^{NEG} neurons consistently show higher magnitude of transcriptional changes compared to HET^{POS} neurons across most cell types. Statistical significance was determined by Wilcoxon rank-sum test. $*p < 0.05$, $**p < 0.01$, $***p < 0.001$, ns = not significant.

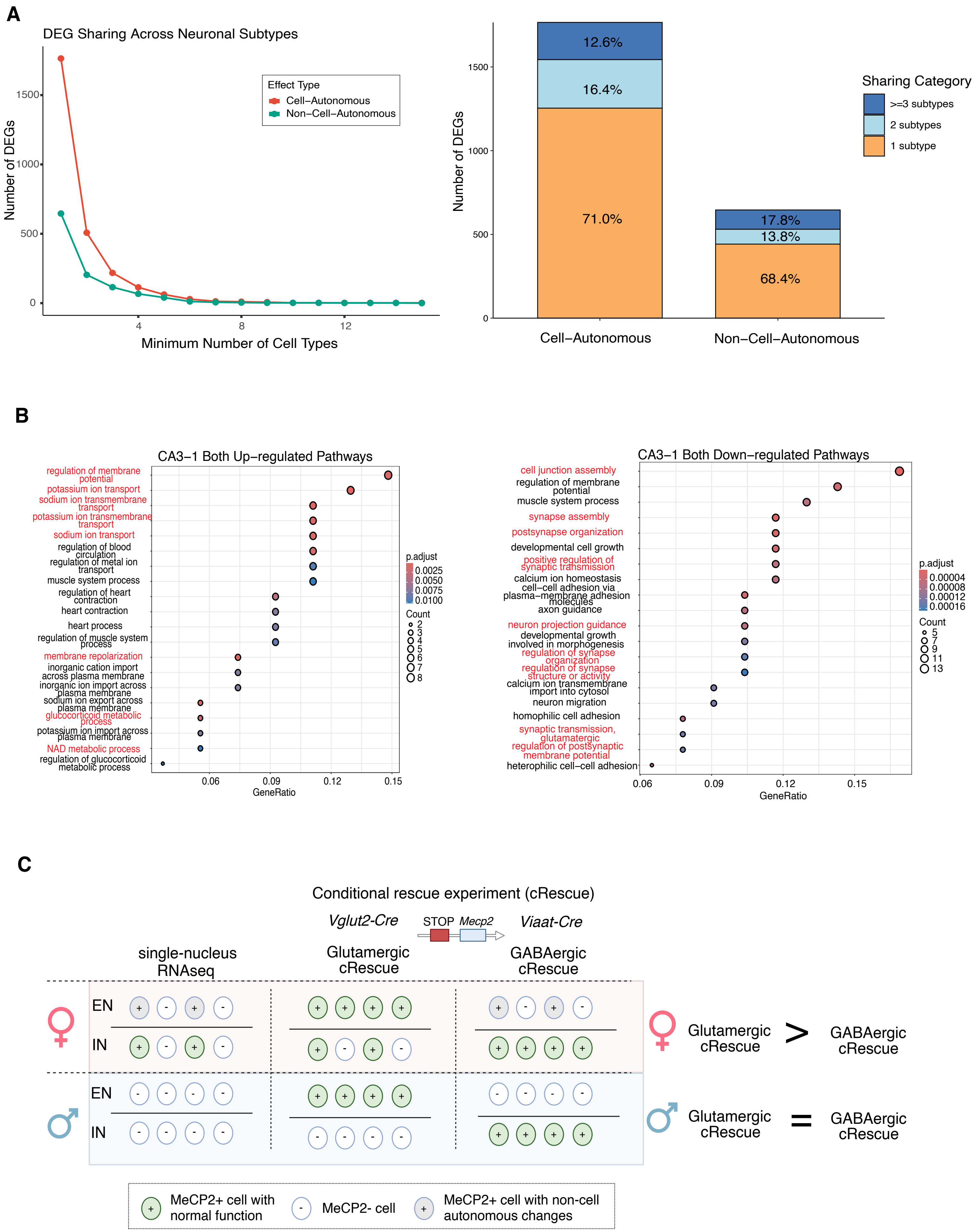
A
DEG Sharing Across Neuronal Subtypes
Effect Type
Cell−Autonomous
Non−Cell−Autonomous
Number of DEGs
Minimum Number of Cell Types
12.6%
16.4%
71.0%
17.8%
13.8%
68.4%
Sharing Category
>=3 subtypes
2 subtypes
1 subtype
Cell−Autonomous
Non−Cell−Autonomous
B
CA3−1 Both Up−regulated Pathways
regulation of membrane potential
potassium ion transport
sodium ion transmembrane transport
potassium ion transmembrane transport
sodium ion transport
regulation of blood circulation
regulation of metal ion transport
muscle system process
regulation of heart contraction
heart contraction
heart process
regulation of muscle system process
membrane repolarization
inorganic cation import across plasma membrane
inorganic ion import across plasma membrane
sodium ion export across plasma membrane
glucocorticoid metabolic process
potassium ion import across plasma membrane
NAD metabolic process
regulation of glucocorticoid metabolic process
p.adjust
Count
GeneRatio
CA3−1 Both Down−regulated Pathways
cell junction assembly
regulation of membrane potential
muscle system process
synapse assembly
postsynapse organization
developmental cell growth
positive regulation of synaptic transmission
calcium ion homeostasis
cell−cell adhesion via plasma−membrane adhesion molecules
axon guidance
neuron projection guidance
developmental growth involved in morphogenesis
regulation of synapse organization
regulation of synapse structure or activity
calcium ion transmembrane import into cytosol
neuron migration
homophilic cell adhesion
synaptic transmission, glutamatergic
regulation of postsynaptic membrane potential
heterophilic cell−cell adhesion
p.adjust
Count
GeneRatio
C
Conditional rescue experiment (cRescue)
Vglut2-Cre
STOP Mecp2
Viaat-Cre
single-nucleus RNAseq
Glutamergic cRescue
GABAergic cRescue
EN
IN
EN
IN
Glutamergic cRescue
>
GABAergic cRescue
Glutamergic cRescue
=
GABAergic cRescue
MeCP2+ cell with normal function
MeCP2- cell
MeCP2+ cell with non-cell autonomous changes

Fig. S5. GO analysis of shared CA3 snDEGs in mosaic RTT and graphic illustration of cell-type specific scenarios as related to conditional rescue experiments in male and female RTT mice. (**A**) Number of DEGs are shared across number of cell types (Left) and stacked bar plots with percent of DEGs that are shared across 3 and more cell types, 2 cell types and unique to 1 cell type. (B) GO pathways enriched in shared genes that are upregulated (Left) or 10downregulated (Right) in both HETPOS and HETNEG in the CA3-1 cluster. (**C**) A working model placing our snRNA-seq findings in the context of previous sex-specific differences observed in conditional rescue experiments in male vs female RTT mice. In the mosaic female RTT mice, re- expressing *Mecp2* in glutamatergic, excitatory neurons rescued neurological phenotypes better than re-expressing *Mecp2* in inhibitory, GABAergic neurons. Our snRNA-seq results show that glutamatergic neurons are particularly disrupted in the mosaic RTT brain, where both MeCP2+ and MeCP2- cells display altered transcriptional responses, compared to the GABAergic neurons where only MeCP2- cells are affected. Therefore, restoring *Mecp2* in glutamatergic neurons rescues both non-cell-autonomous and cell-autonomous changes (i.e. a larger proportion of neurons) than only rescuing cell-autonomous changes in GABAergic interneurons. In male RTT models, re-expressing *Mecp2* in either glutamatergic or GABAergic neurons had similar levels of rescue, particularly on survival. Graphical illustration created using BioRender.com.

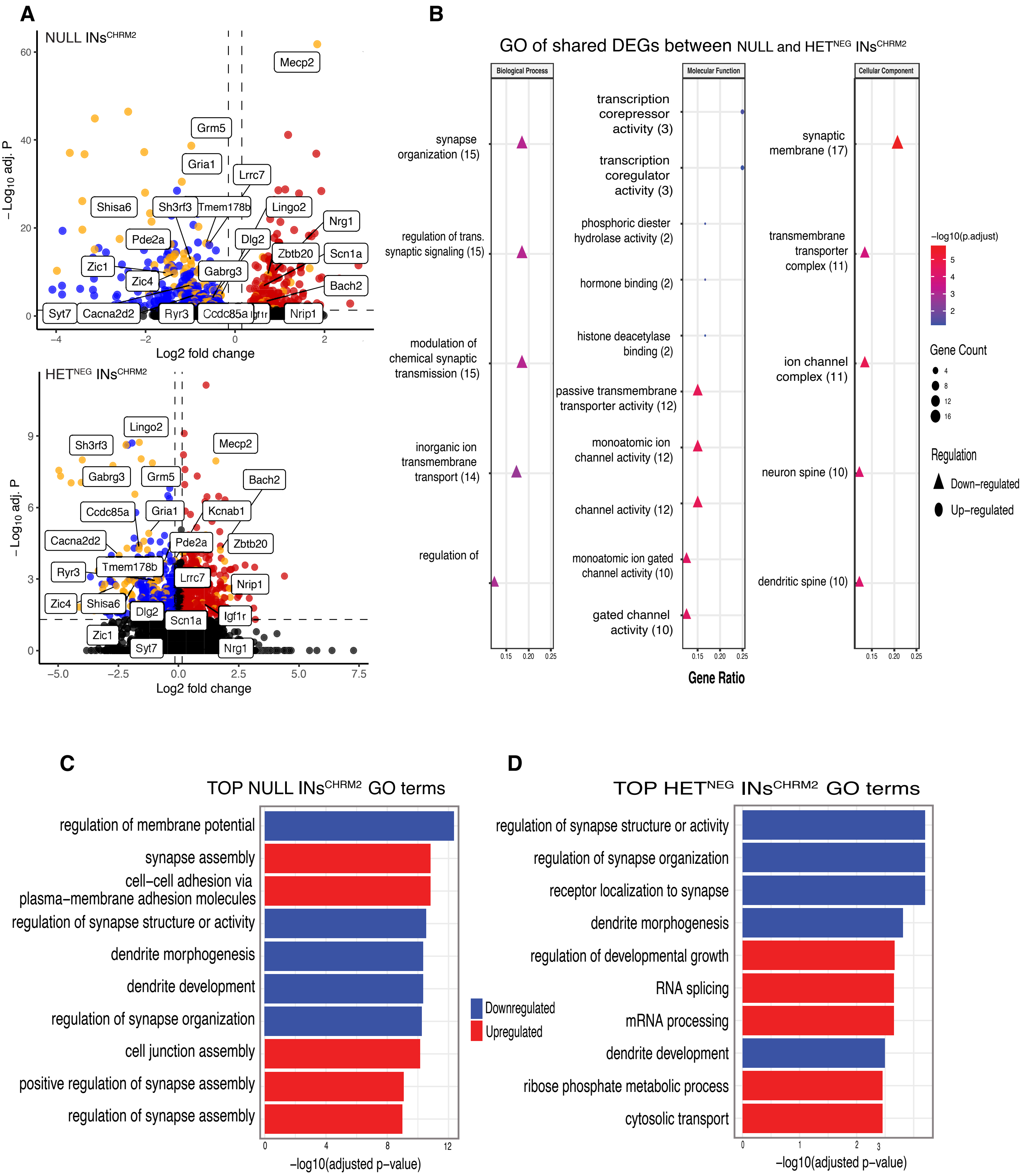
A
NULL INsCHRM2
HETNEG INsCHRM2
−Log10 adj. P
Log2 fold change
Mecp2
Grm5
Gria1
Lrrc7
Shisa6
Sh3rf3
Tmem178b
Lingo2
Nrg1
Pde2a
Dlg2
Zbtb20
Scn1a
Zic1
Gabrg3
Zic4
Bach2
Syt7
Cacna2d2
Ryr3
Ccdc85a
Igf1r
Nrip1
Kcnab1
B
GO of shared DEGs between NULL and HETNEG INsCHRM2
Biological Process
Molecular Function
Cellular Component
synapse organization (15)
regulation of trans. synaptic signaling (15)
modulation of chemical synaptic transmission (15)
inorganic ion transmembrane transport (14)
regulation of
transcription corepressor activity (3)
transcription coregulator activity (3)
phosphoric diester hydrolase activity (2)
hormone binding (2)
histone deacetylase binding (2)
passive transmembrane transporter activity (12)
monoatomic ion channel activity (12)
channel activity (12)
monoatomic ion gated channel activity (10)
gated channel activity (10)
synaptic membrane (17)
transmembrane transporter complex (11)
ion channel complex (11)
neuron spine (10)
dendritic spine (10)
Gene Ratio
−log10(p.adjust)
Gene Count
Regulation
Down−regulated
Up−regulated
C
TOP NULL INsCHRM2 GO terms
regulation of membrane potential
synapse assembly
cell−cell adhesion via plasma−membrane adhesion molecules
regulation of synapse structure or activity
dendrite morphogenesis
dendrite development
regulation of synapse organization
cell junction assembly
positive regulation of synapse assembly
regulation of synapse assembly
−log10(adjusted p−value)
Downregulated
Upregulated
D
TOP HETNEG INsCHRM2 GO terms
regulation of synapse structure or activity
regulation of synapse organization
receptor localization to synapse
dendrite morphogenesis
regulation of developmental growth
RNA splicing
mRNA processing
dendrite development
ribose phosphate metabolic process
cytosolic transport
−log10(adjusted p−value)

Fig. S6. **Volcano plots and GO analysis of trilaminar interneurons.** (**A**) Volcano plot of snDEGs (up-regulated-red, down-regulated-blue) in the trilaminar interneurons (INsCHRM2) of the NULL (top) HETNEG (bottom), highlighting shared DEGs in orange between the two datasets. (**B**) GO pathways enriched in DEGs that are shared (both upregulated and down downregulated) between trilaminar interneurons populations from NULL and HETNEG samples. (**C**) Top GO pathways for NULL Chrm2+ INs (**D**) Top GO pathways for HET-NEG Chrm2+ INs.

Supplemental tables:

Table S1. Differential expressed genes from bulk RNAseq.
Table S2. Top marker genes for each cluster in snRNAseq.
Table S3. Number of nuclei in each cluster in the snRNAseq.
Table S4. Differential expressed genes from snRNAseq.
Table S5. Hypergeometric enrichment analysis of gene overlaps between experimental conditions.
Table S6. Hypergeometric enrichment analysis of overlapped DEGs between HET-POS and HET-NEG neurons.
Table S7. Wild-type cell subsampling validation of false positive rates.
Table S8. GO analysis for each cluster compared between HET-POS and HET-NEG.
